# Progesterone Enhances Niraparib Efficacy in Ovarian Cancer by Promoting Palmitoleic-Acid-Mediated Ferroptosis

**DOI:** 10.34133/research.0371

**Published:** 2024-05-24

**Authors:** Nayiyuan Wu, Xiu Zhang, Chao Fang, Miaochen Zhu, Zhibin Wang, Lian Jian, Weili Tan, Ying Wang, He Li, Xuemeng Xu, Yujuan Zhou, Tang-Yuan Chu, Jing Wang, Qianjin Liao

**Affiliations:** ^1^ The Affiliated Cancer Hospital of Xiangya School of Medicine, Central South University/Hunan Key Laboratory of Cancer Metabolism, Hunan Cancer Hospital, Changsha 410078, Hunan, China.; ^2^ Public Service Platform of Tumor Organoids Technology, Hunan Gynecological Tumor Clinical Research Center, Changsha 410013, Hunan, China.; ^3^ Hunan Key Laboratory of the Research and Development of Novel Pharmaceutical Preparations, Changsha Medical University, Changsha 410219, Hunan, China.; ^4^ Department of Obstetrics & Gynecology, Hualien Tzu Chi Hospital, Buddhist Tzu Chi Medical Foundation, Hualien 970, Taiwan, China.

## Abstract

Poly (adenosine 5′-diphosphate-ribose) polymerase inhibitors (PARPi) are increasingly important in the treatment of ovarian cancer. However, more than 40% of *BRCA1/2-*deficient patients do not respond to PARPi, and *BRCA* wild-type cases do not show obvious benefit. In this study, we demonstrated that progesterone acted synergistically with niraparib in ovarian cancer cells by enhancing niraparib-mediated DNA damage and death regardless of *BRCA* status. This synergy was validated in an ovarian cancer organoid model and in vivo experiments. Furthermore, we found that progesterone enhances the activity of niraparib in ovarian cancer through inducing ferroptosis by up-regulating palmitoleic acid and causing mitochondrial damage. In clinical cohort, it was observed that progesterone prolonged the survival of patients with ovarian cancer receiving PARPi as second-line maintenance therapy, and high progesterone receptor expression combined with low glutathione peroxidase 4 (GPX4) expression predicted better efficacy of PARPi in patients with ovarian cancer. These findings not only offer new therapeutic strategies for PARPi poor response ovarian cancer but also provide potential molecular markers for predicting the PARPi efficacy.

## Introduction

Epithelial ovarian cancer (EOC) generally presents at an advanced stage and is the most common cause of gynecological cancer death [1,2]. Poly (adenosine 5′-diphosphate-ribose) polymerase inhibitors (PARPi) have significantly improved maintenance therapy for recurrent ovarian cancer and are now approved as a first-line treatment for women with breast cancer susceptibility gene 1/2 (BRCA1/2) mutations [3–14]. However, only about 25% of patients with ovarian cancer have *BRCA* mutations [15,16]. Patients with BRCA wild-type ovarian cancer, especially those with homologous recombination deficiency (HRD)-negative tumors, do not benefit as much from PARPi [17–20], which limits their use in ovarian cancer therapy. In addition, except for the difference between BRCAness/PARPi sensitivity and non-BRCAness/PARPi resistance, EOCs (including type 1 and type 2 EOCs) are composed of distinct histological subtypes with unique genomic characteristics, resulting in inconsistent responses to PARPi [21–23]. Type 2 EOCs, including high-grade serous carcinoma (HGSC), carcinosarcoma, and undifferentiated carcinoma, are typically characterized by mutations in genes such as tumor protein P53 (*TP53*) and *BRCA1/2*. Type 1 EOCs, including several subtypes such as low-grade serous ovarian carcinoma, mucinous adenocarcinoma, endometrioid carcinoma, and clear cell carcinoma, are typically characterized by a specific pattern of mutations in genes such as *KRAS*, *BRAF*, and *PTEN*, which response poorly to PARPi. Therefore, improving the efficacy of PARPi in patients with ovarian cancer, especially non-BRCAness HGSC and type 1 EOC, is urgently required.

Niraparib, a PARPi, is approved for maintenance therapy in recurrent ovarian cancer, regardless of BRCA mutation status [13,24]. The PRIMA/PRIME and NOVA/NORA clinical studies demonstrated significant clinical efficacy of niraparib in patients with BRCA1/2 mutant ovarian cancer. However, its efficacy was reduced in patients with wild-type BRCA, particularly those with wild-type BRCA/HRD-negative status [13,24–26]. In addition, both the NOVA/NORA and PRIMA/PRIME studies found that niraparib causes adverse reactions in the digestive and vascular systems of patients with ovarian cancer. In the NOVA study, 14.7% of patients discontinued the treatment, 68.9% interrupted the treatment, and 66.5% reduced drug dosage due to side effects; after 4 months, only 25% of patients continued the treatment at the initial dose. Consequently, the de-escalation of niraparib maintenance therapy while preserving clinical efficacy is crucial. Combination therapy with PARPi is urgently needed to address this challenge [27,28].

Progesterone (P4), a natural hormone produced by the ovary, is commonly used to treat well-differentiated EOC and endometrial cancer [29–34]. Epidemiological studies have shown that P4 can prevent the occurrence of ovarian cancer [35–40]. Our previous studies demonstrated that P4 could eliminate the precancerous cells in the fallopian tube either by inducing necroptosis through accelerating intracellular reactive oxygen species (ROS) and DNA damage [41] or by inducing pyroptosis through the paracrine inflammatory effect exerted by the adjacent fibroblasts [42]. Notably, intracellular ROS levels and DNA damage are closely associated with tumor cell sensitivity to PARPi [43,44], suggesting that P4 might improve the efficacy of niraparib in the treatment of ovarian cancer.

In this study, we investigated the therapeutic effect PARPi in combination with P4 in in vitro and in vivo cell models of type 1 and type 2 EOCs, as well as in organoid model of patients with HGSC. We demonstrated that P4 synergistically enhances the therapeutic activity of niraparib by inducing niraparib-mediated DNA damage in both type 1 and type 2 EOC cells regardless of the *BRCA*/HRD. We further demonstrated that P4 induced ferroptosis by promoting lipid oxidation and palmitoleic acid (POA) generation. Notably, P4 prolonged the survival of patients with ovarian cancer receiving PARPi as second-line maintenance therapy. In addition, P4 receptor (PR)-high/glutathione peroxidase 4 (GPX4)-low expression predicted better efficacy of PARPi and longer outcomes in patients with HGSC.

## Results

### P4 sensitizes ovarian cancer cells to niraparib by enhancing niraparib-mediated DNA damage and apoptosis

**Fig. 1.**
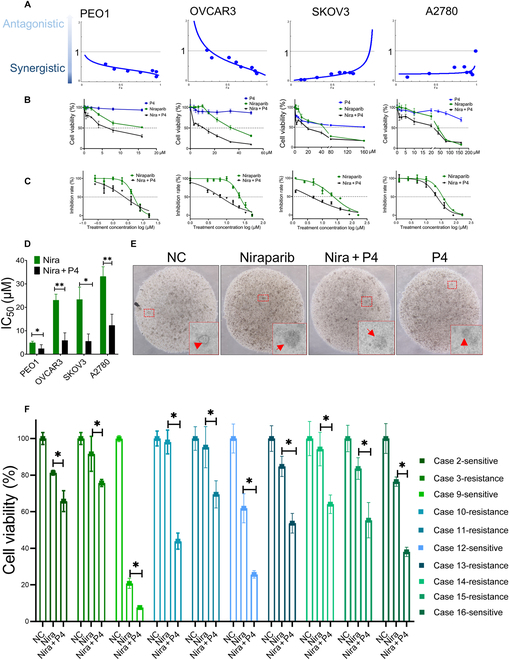
P4 enhances the inhibitory effect of niraparib in ovarian cancer. (A) P4 plus niraparib combination treatment at a ratio of 1:1 in human *BRCA*2-mutated ovarian cancer cell line PEO1 and wild-type *BRCA* ovarian cancer cell lines OVCAR3, SKOV3, and A2780. (B) Cell counting kit-8 (CCK8) test cell viability in PEO1, OVCAR3, SKOV3, and A2780 cells treated with different concentration gradients of vehicle (NC), P4 (blue line), niraparib (green line), and P4 plus niraparib (black line) for 48 h. (C and D) half inhibitory concentration (IC_50_) of PEO1, OVCAR3, SKOV3, and A2780 cells. (E) Representative images of ovarian cancer organoid tissues after the above treatment with vehicle, niraparib, P4 plus niraparib, or P4 for 5 d, acquired by light microscopy. (F) Cell viabilities in 10 ovarian cancer organoid tissue cases were tested after treatment with vehicle, niraparib, P4 plus niraparib or P4 for 5 d. *P < 0.05; **P < 0.01.

We selected 4 EOC cell lines in this study, including 2 type 2 HGSC cells—*BRCA* wild-type (*BRCA*^WT^) OVCAR3 and *BRCA2*-mutated (*BRCA2*^Mut^) PEO1—and 2 type 1 endometroid/clear cells—A2780 and SKOV3. PEO1 and OVCAR3 cells were treated with different concentrations of P4 plus niraparib (niraparib:P4 = 1:10, 1:6, 1:4, 1:2, 1:1, and 1:0.5) for 48 h. Synergetic effects [median effective dose (ED50) < 1] were observed for P4 plus niraparib at concentration ratios of niraparib:P4 between 1:4 and 1:0.5; the best combination indexes (CI) was found at niraparib:P4 = 1:1 in SKOV3, A2780, and PEO1 cells (Fig. 1A, Table 1, and Fig. S1A and B). Niraparib-induced growth inhibition (green line) was significantly enhanced by the P4 combined treatment (black line) in all 4 ovarian cancer cell lines tested (Fig. 1B).

**Table 1.** Synergistic effect of P4 plus niraparib at different ratios

Ratio (niraparib:P4)	1:10	1:6	1:4	1:2	1:1	1:0.5
PEO1 CI (ED_50_)	1.02	0.92	0.91	0.45	0.38	0.45
OVCAR3 CI (ED_50_)	1.26	1.34	0.56	0.33	0.45	0.01
SKOV3 CI (ED_50_)	–	–	–	0.21	0.19	0.34
A2780 CI (ED_50_)	–	–	–	0.53	0.25	0.41

0.9 ≤ CI ≤ 1.1, the combination of the 2 drugs has superposition effect; 0.8 ≤ CI < 0.9, the combination of the 2 drugs has slight synergistic effect; 0.6 ≤ CI < 0.8, the combination of the 2 drugs has moderate synergistic effect; 0.4 ≤ CI < 0.6, the combination of the 2 drugs has satisfied synergistic effect; 0.2 ≤ CI < 0.4, the combination of the 2 drugs has great synergistic effect.

Moreover, P4 sensitized cancer cells to niraparib reflected by lower IC_50_ values for niraparib in OVCAR3, SKOV3, A2780, and PEO1 cells (Fig. 1C and D). Further, compared with the *BRCA2*-mutated cell line PEO1, P4 significantly reduced IC_50_ in wild-type *BRCA* cell lines (OVCAR3, SKOV3, and A2780); the IC_50_ of OVCAR3 was reduced from 23.07 (20.79 to 25.60) μM to 5.903 (3.816 to 9.131) μM, the IC_50_ of SKOV3 was reduced from 23.39 (16.78 to 32.59) μM to 5.580 (2.671 to 11.65) μM, and the IC_50_ of A2780 was reduced from 33.22 (29.55 to 37.34) μM to 12.340 (7.978 to 19.08) μM, while the IC_50_ of PEO1 was reduced from 4.953 (4.414 to 5.558) μM to 2.410 (1.422 to 4.084) μM (Fig. 1D). Importantly, the ovarian cancer organoid model confirmed the synergistic effects of P4 and niraparib regardless of BRCA, and 10 HGSC cases were included. P4 combined with niraparib exerted satisfactory synergistic effects (ED_50_ < 1) and significantly inhibited cell viability in both niraparib-sensitive and -resistant cases (Fig. 1E and F and Table 2).

**Table 2.** Synergistic effect of niraparib plus P4 in organoid tissue

Case	CI(ED_50_)	Platinum sensitivity	Niraparib sensitivity	HRD status	BRCA status
Case 2	0.55	–	Sensitive	–	–
Case 3	0.73	Sensitive	Resistance	–	BRCA2 mutation
Case 9	1.02	Resistance	Sensitive	Positive	Wild
Case 10	0.61	Resistance	Resistance	Positive	Wild
Case 11	0.65	Sensitive	Resistance	–	–
Case 12	0.54	Sensitive	Sensitive	–	–
Case 13	0.78	Resistance	Resistance	–	Wild
Case 14	0.72	Resistance	Resistance	–	Wild
Case 15	0.68	Sensitive	Resistance	–	–
Case 16	0.47	Sensitive	Sensitive	Positive	BRCA1 mutation

0.9 ≤ CI ≤ 1.1, the combination of the 2 drugs has superposition effect; 0.8 ≤ CI < 0.9, the combination of the 2 drugs has slight synergistic effect; 0.6 ≤ CI < 0.8, the combination of the 2 drugs has moderate synergistic effect; 0.4 ≤ CI < 0.6, the combination of the 2 drugs has satisfied synergistic effect

**Fig. 2.**
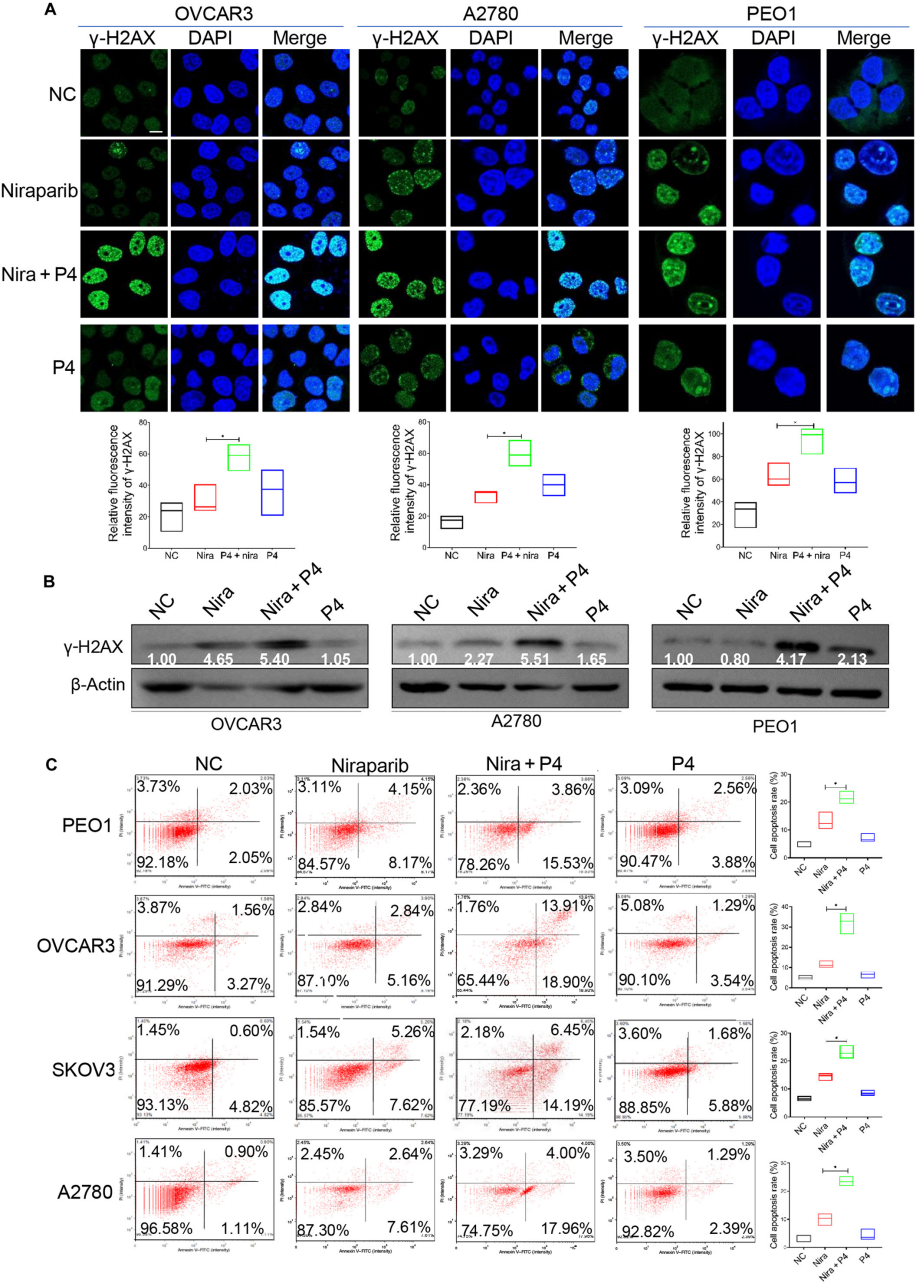
P4 enhances the sensitivity of ovarian cancer cells to niraparib by inducing DNA damage and apoptosis. Ovarian cancer cells were treated with vehicle (NC), niraparib, P4 (10 μM) plus niraparib (10 μM), or P4 (10 μM) for 24 h, and IF (A) and WB (B) were performed to assess γ-H2AX expression. (C) Flow cytometry test cell death rate. The data in the graph represent annexin-V-positive cells. *P < 0.05. Scale bar, 10 μM. FITC, fluorescein isothiocyanate.

Clone formation, cell scratching, and Transwell assays were conducted on *BRCA*^WT^ and *BRCA2*^Mut^ ovarian cancer cells. Combined treatment with P4 and niraparib resulted in a substantial reduction in the number and dimensions of colonies, as well as a diminution in cellular invasion and metastatic potential (Fig. S1C to E) in both *BRCA*^WT^ and *BRCA2*^Mut^ cells compared with either niraparib or P4 monotherapy. Immunohistochemistry (IHC) and Western blot (WB) analyses demonstrated that the combined treatment with P4 and niraparib significantly elevated the levels of gamma histone family member X (γ-H2AX) compared to niraparib alone (Fig. 2A and B). The unrepaired double-strand break induced by this combinatorial approach triggered apoptosis, leading to a more pronounced apoptotic response compared to either agent administered independently in ovarian cancer cells (Fig. 2C). Collectively, these findings suggest that P4 potentiates the sensitivity of ovarian cancer cells to niraparib by exacerbating DNA double-strand break and inducing severe apoptosis.

### P4 enhances the antitumor effect of niraparib prolonged survival in vivo

**Fig. 3.**
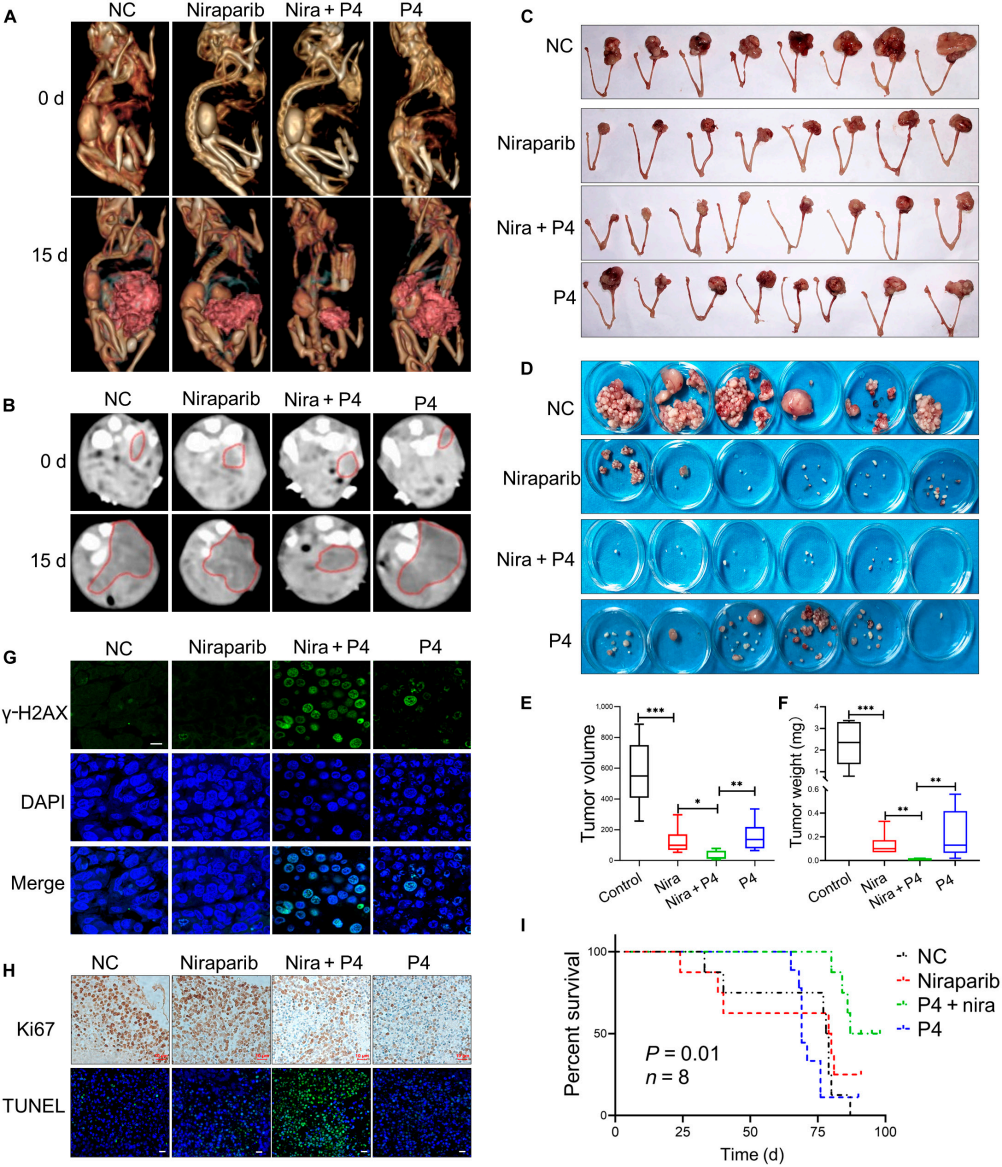
P4 enhances the efficacy of niraparib in ovarian cancer in vivo. Representative 3-dimensional CT images (A) and CT scans (B) of ovarian in situ tumorigenesis mice before treatment (0 d) and 15 d after drug administration. (C and E) Ovarian in situ tumorigenesis mice and (D and F) intraperitoneal tumorigenesis mice treated with vehicle, niraparib (50 mg/kg), P4 plus niraparib (P4 at 5 mg/kg and niraparib at 50 mg/kg), or P4 (5 mg/kg); mice were sacrificed after 28 d of drug administration, and tumor sizes and weights were obtained. (G) Immunofluorescence (IF) test γ-H2AX expression in mouse tumor tissue samples; (H) IHC of the cell-proliferation-associated antigen Ki67 and the terminal-deoxynucleotidyl-transferase-mediated deoxyuridine triphosphate nick end labeling (TUNEL) test for apoptosis-related markers in the mouse tumor tissue. (I) Kaplan–Meier survival analysis of the survival of C57BL/6 mice in 4 different treatment groups (*n* = 8). *P < 0.05; **P < 0.01, ***P < 0.001. Scale bars,10 μm.

The peritoneal tumorigenesis mouse model and ovarian in situ tumorigenesis mouse model were established to confirm the synergistic effects of P4 and niraparib in vivo. In the ovarian in situ tumorigenesis model, after 15 d of administration of the drug, computed tomography (CT) images showed that ovarian tumor volumes were smaller in the P4 + niraparib combination group compared with the niraparib alone group. Representative images were shown in Fig. 3A and B. Ovarian tumor volumes were smaller in the combination group compared with the niraparib alone group in both the ovarian in situ (Fig. 3C and E) and peritoneal (Fig. 3D and F) tumorigenesis mouse models**.** In the mouse tumor tissue, IF showed higher γ-H2AX expression in the P4 + niraparib group compared with niraparib alone (Fig. 3G). In addition, the cell-proliferation-associated antigen Ki67 was suppressed, and apoptosis-related markers were induced in the P4 + niraparib group compared with the niraparib alone cohort (Fig. 3H). Survival experiments showed that the P4 + niraparib group significantly prolonged the survival time compared with niraparib alone in C57BL/6j mice (Fig. 3I). In vivo experiments confirmed the in vitro data, with P4 enhancing niraparib antitumor activity by inducing niraparib-mediated DNA damage and apoptosis in ovarian cancer cells.

### P4 enhances the activity of niraparib in ovarian cancer by increasing fatty acid metabolism and POA production

**Fig. 4.**
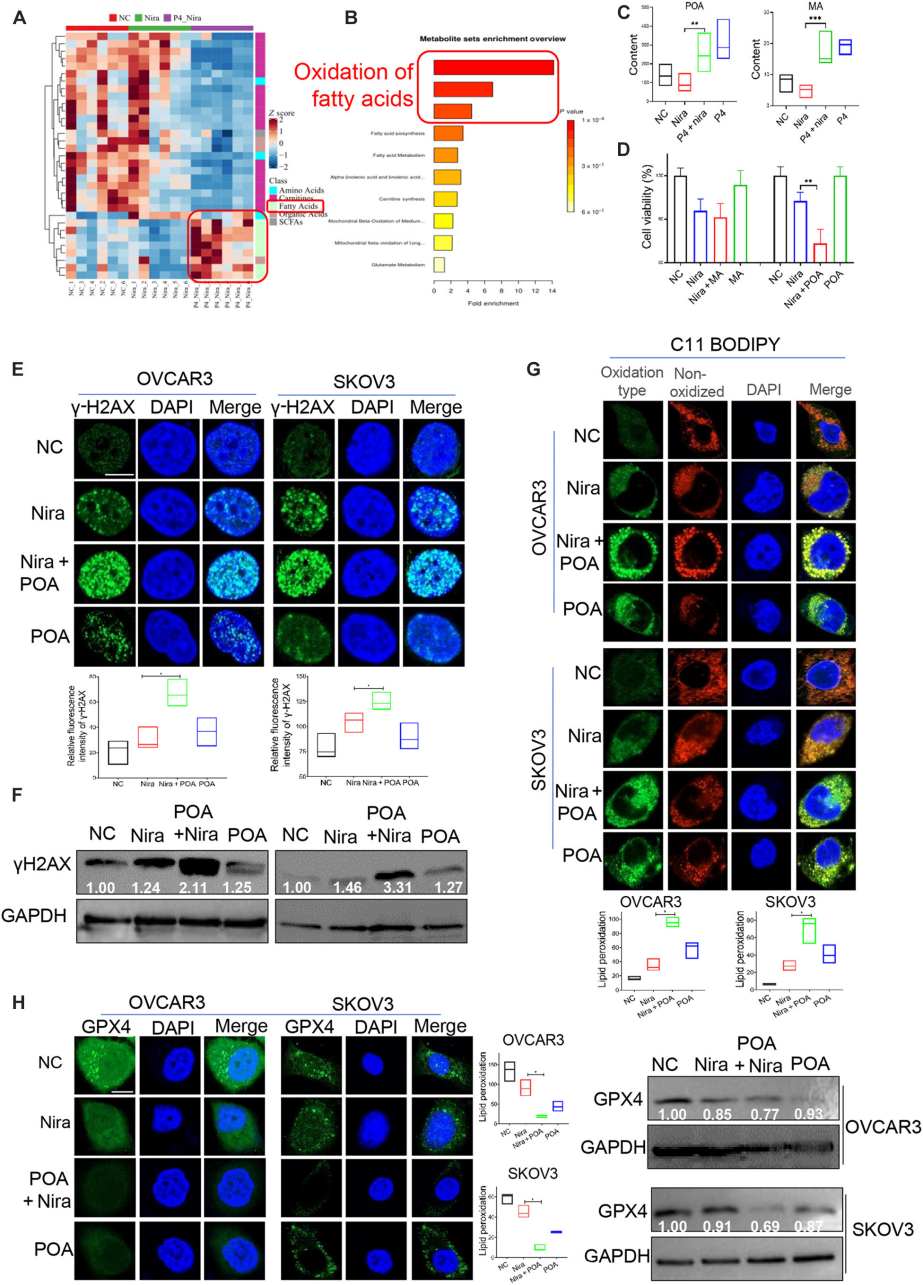
P4 enhances the activity of niraparib by up-regulating fatty acid metabolism and POA production in ovarian cancer cells. (A) Metabolomics were performed in OVCAR3 cells after treated with vehicle (NC), niraparib, P4 plus niraparib, or P4 for 24 h; the *Z* score heatmap of the overall profile of metabolites. (B) Enrichment analysis results of metabolomics. (C) Detection of POA and MA in OVCAR3 cells after 24 h after the above treatment. (D) CCK8 assay tested the viability of OVCAR3 cells after 24 h of treatment with vehicle, niraparib, POA or MA + niraparib, POA, or MA. (E) IF and (F) WB were performed to assess the expression of γ-H2AX in OVCAR3 and SKOV3 cells after treatment with vehicle, niraparib (10 μM), POA (200 μM) + niraparib (10 μM), or POA (200 μM) for 24 h. (G) BODIPY 581/591 C11 staining was performed to quantify lipid peroxidation in OVCAR3 administered with the above treatment. (H) IF and WB were performed to assess GPX4 expression in OVCAR3 and SKOV3 cells administered with the above treatment. *P < 0.05; **P < 0.01; ***P < 0.001. Scale bar, 10 μm.

To further explore the mechanisms of P4 promoting the inhibitory effect of niraparib on ovarian cancer, we performed metabolomics analysis, revealing that fatty acid oxidation metabolism were significantly enhanced in the niraparib plus P4 group compared with niraparib treatment alone (Fig. 4A and B). Quantitative metabolomics also demonstrated that production of POA and myristoleic acid (MA) was increased in the P4 plus niraparib group compared with the niraparib alone group (Fig. 4C). The CCK8 assay showed that POA, not MA, enhanced the inhibitory effect of niraparib in ovarian cancer cells (Fig. 4D). POA enhanced niraparib’s antitumor activity by inducing niraparib-mediated DNA damage, and combined treatment with POA and niraparib significantly increased the levels of γ-H2AX (Fig. 4E and F).

To determine how P4 up-regulates POA in ovarian cancer cells, we performed transcriptomic sequencing of OVCAR3 cells. Results showed that the lipid metabolism signaling pathway was significantly enhanced in the combination therapy group (Fig. S2A), and multiple fatty-acid-metabolism-related genes were significantly up-regulated (Fig. S2B). To confirm the RNA sequencing results, we selected 10 significantly up-regulated genes involved in fatty acid metabolism (*SCD*, *FASN*, *DHCR7*, *MVD*, *ETV4*, *PER1*, *ACSS2*, *INSIG1*, *PSCK9*, and *FDFT1*) and analyzed those by quantitative real-time polymerase chain reaction (PCR) in OVCAR3, SKOV3, A2780, and PEO1 cells (Fig. S2C and Table S1). Treatment with P4 combined with niraparib significantly increased the expression of stearoyl-coenzyme A desaturase 1 (SCD1) compared with niraparib alone in ovarian cell lines (Fig. S2C and D). SCD1 inhibitors rescued the inhibitory effect of P4 plus niraparib on ovarian cancer cells (Fig. S2E). SCD1 was the rate-limiting enzyme required for the production of monounsaturated fatty acids from saturated fatty acids, such as converting palmitic acid (16) to POA (16, 1) [45,46]. This suggests that P4 up-regulates fatty acid metabolism and POA production by the expression of up-regulating fatty-acid-metabolism-related genes (SCD1, etc.).

How P4 and niraparib combination up-regulates SCD1 was further explored. We predicted the transcription factors of the *SCD* promoter via http://genome.ucsc.edu/. It was shown that *SCD* promoter could be bound by PR. In addition, PR showed significant nuclear translocation in the P4 and niraparib combination group (Fig. S3A). Chromatin immunoprecipitation assay provides direct evidence for PR binding to SCD gene promoter in this study (Fig. S3B and C); the sterol regulatory element-binding protein 1 (SREBP1), which was identified as positive effectors of SCD transcription [47], was selected as positive control. This suggests that PR may act as a positive effector of SCD transcription.

### P4 enhances the activity of niraparib in ovarian cancer by promoting ferroptosis

**Fig. 5.**
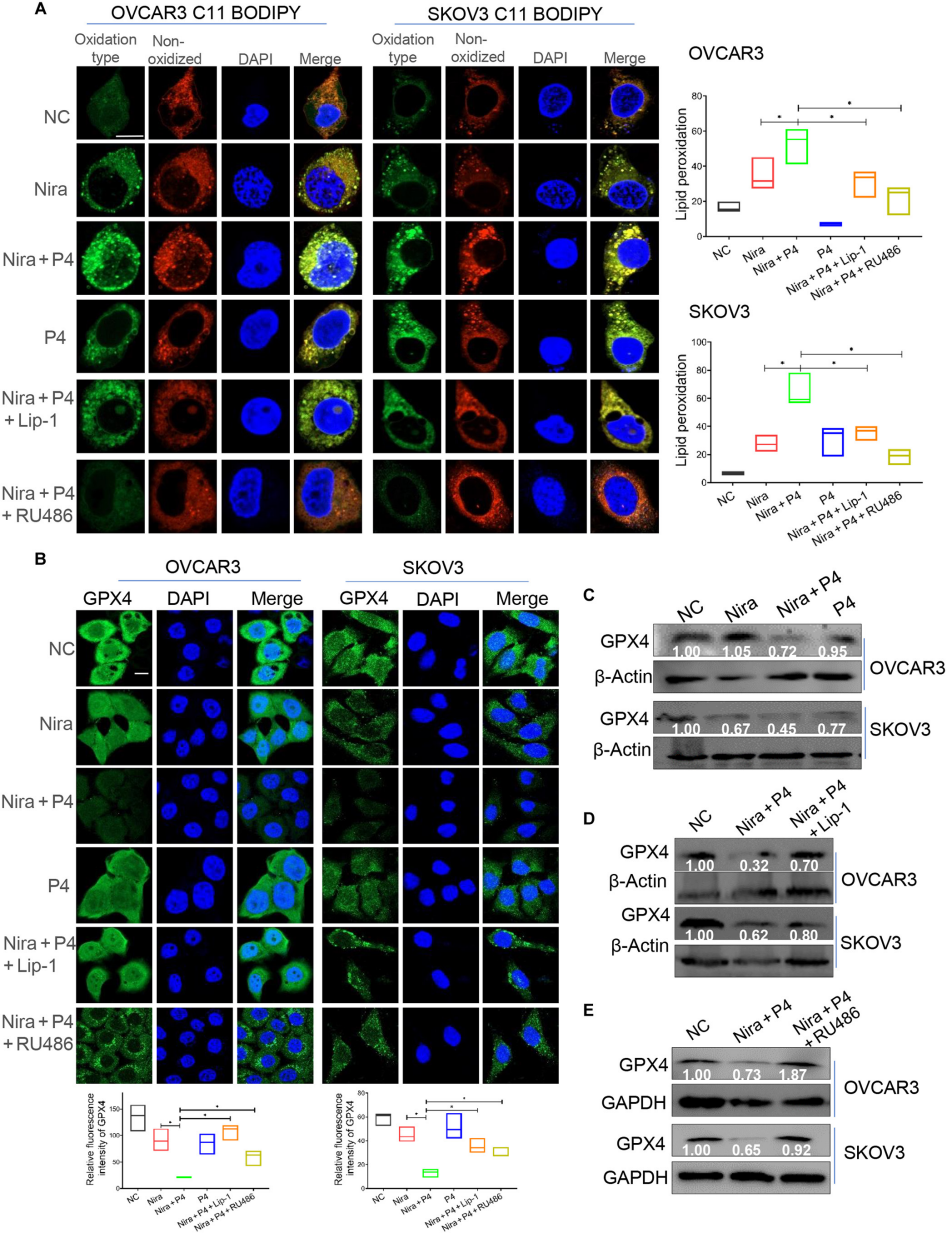
P4 combined with niraparib promote lipid oxidation and GPX4 suppression. (A) BODIPY 581/591 C11 staining was performed to quantify lipid peroxidation in OVCAR3 and SKOV3 cells treated with vehicle (NC), P4 + niraparib, P4, P4 + niraparib + liproxstatin-1 (Lip-1) (1 μM), or P4 + niraparib + RU486 (5 μM) for 24 h. (B to E). IF and WB tested GPX4 expression in OVCAR3 and SKOV3 cells administered with the above treatments. *P < 0.05. Scale bars, 10 μm.

**Fig. 6.**
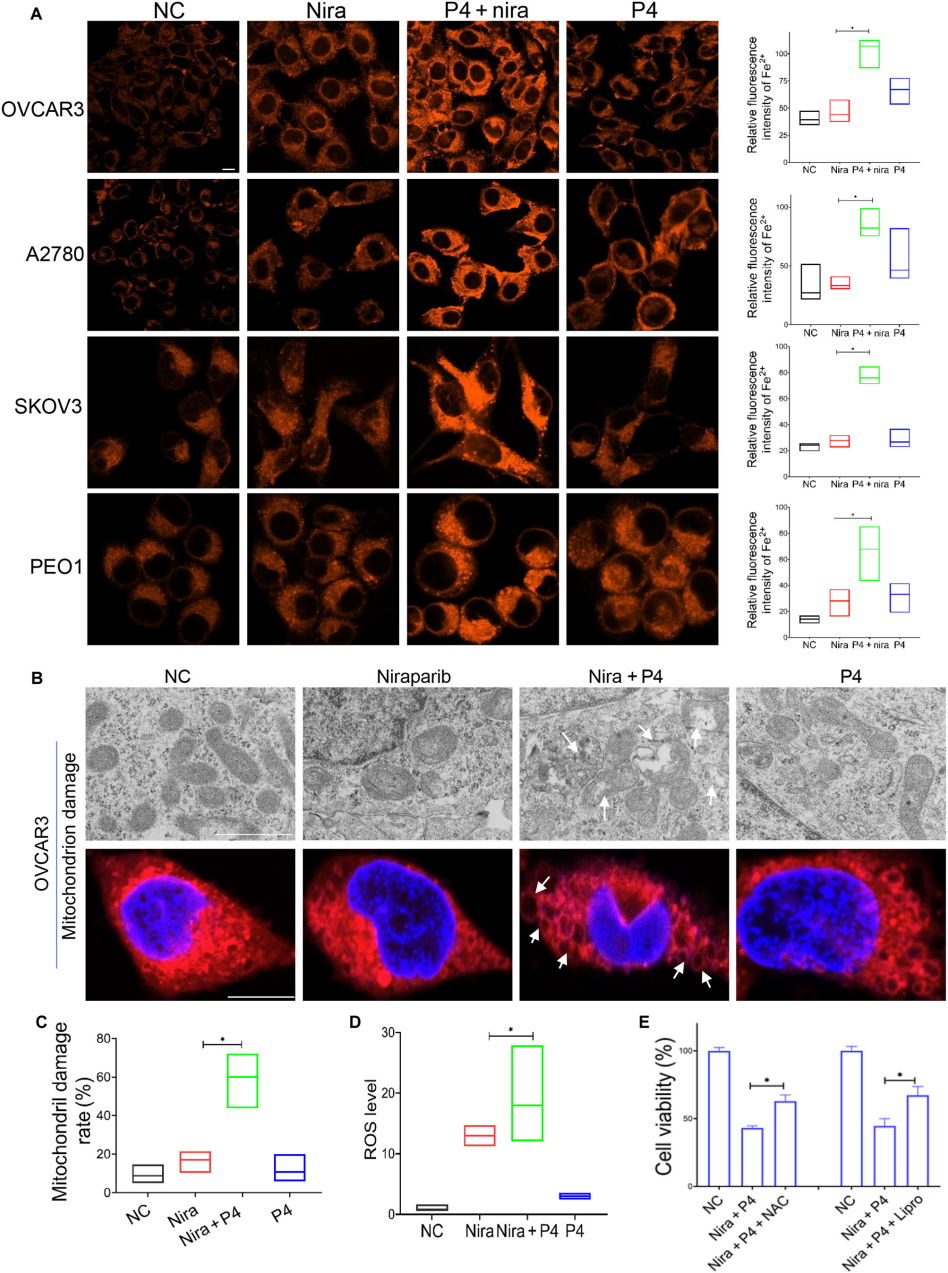
P4 combined with niraparib promote ferroptosis in ovarian cancer cells. (A) IF determined Fe^2+^ levels in OVCAR3, A2780, SKOV3, and PEO1 cells treated with vehicle (NC), niraparib, P4 + niraparib, or P4 for 24 h. (B and C) Transmission electron microscope and IF observed mitochondrial damage of the OVCAR3 cells administered with the above treatments. (D) Flow cytometry test of ROS of OVCAR3 cells administered with the above treatments. (E) CCK8-tested ovarian OVCAR3 cell viability treated with vehicle, P4 + niraparib, P4 + niraparib + NAC, or liproxstatin-1. Scale bars, 10 μm (IF) and 1 μm (transmission electron microscope). *P < 0.05.

The oxidative metabolism of fatty acids, particularly lipid peroxidation, is closely related to ferroptosis [48,49]. In this study, BODIPY 581/591 C11 staining showed that niraparib plus POA markedly induced lipid peroxidation in ovarian cancer cells (Fig. 4G) and inhibited the expression of GPX4, which detoxified phospholipid peroxidation and protected the cells from ferroptosis (Fig. 4H). Similar results were observed in cells treated with P4 in combination with niraparib; treatment with P4 combined with niraparib significantly induced lipid peroxidation in the ovarian cancer cell lines. The potent ferroptosis inhibitor liproxstatin-1 (potent ferroptosis inhibitor) and RU486 (PR inhibitor) abolished the accumulation of lipid peroxides induced by niraparib plus P4 (Fig. 5A). Consistently, niraparib plus P4 significantly inhibited GPX4 expression (Fig. 5B and C and Fig. S4A), and liproxstatin-1 and RU486 rescued the inhibitory effect of P4 plus niraparib on GPX4 expression (Fig. 5B, D, and E). IF and flow cytometry revealed that Fe^2+^ amounts were significantly increased in the combination group compared with niraparib or P4 alone (Fig. 6A and Fig. S4B). P4 combined with niraparib induced severe mitochondrial damage (Fig. 6B and C) and increased ROS levels in OVCAR3 cells compared with niraparib alone (Fig. 6D and Fig. S4C). Mitochondria play important roles in triggering ferroptosis [50], and the generation of mitochondrial ROS is critical for lipid peroxidation and ferroptosis onset. Liproxstatin-1 and the ROS scavenger *N*-acetyl-l-cysteine (NAC) rescued the inhibitory effect of niraparib plus P4 on ovarian cancer cells (Fig. 6E). These data suggested that P4 increased the inhibitory effect of niraparib by promoting ferroptosis through fatty acid metabolism in ovarian cancer.

### P4 enhances PARPi efficacy in ovarian cancer second-line maintenance therapy, and PR-high/GPX4-low expression was linked to better prognosis and higher PARPi sensitivity

**Fig. 7.**
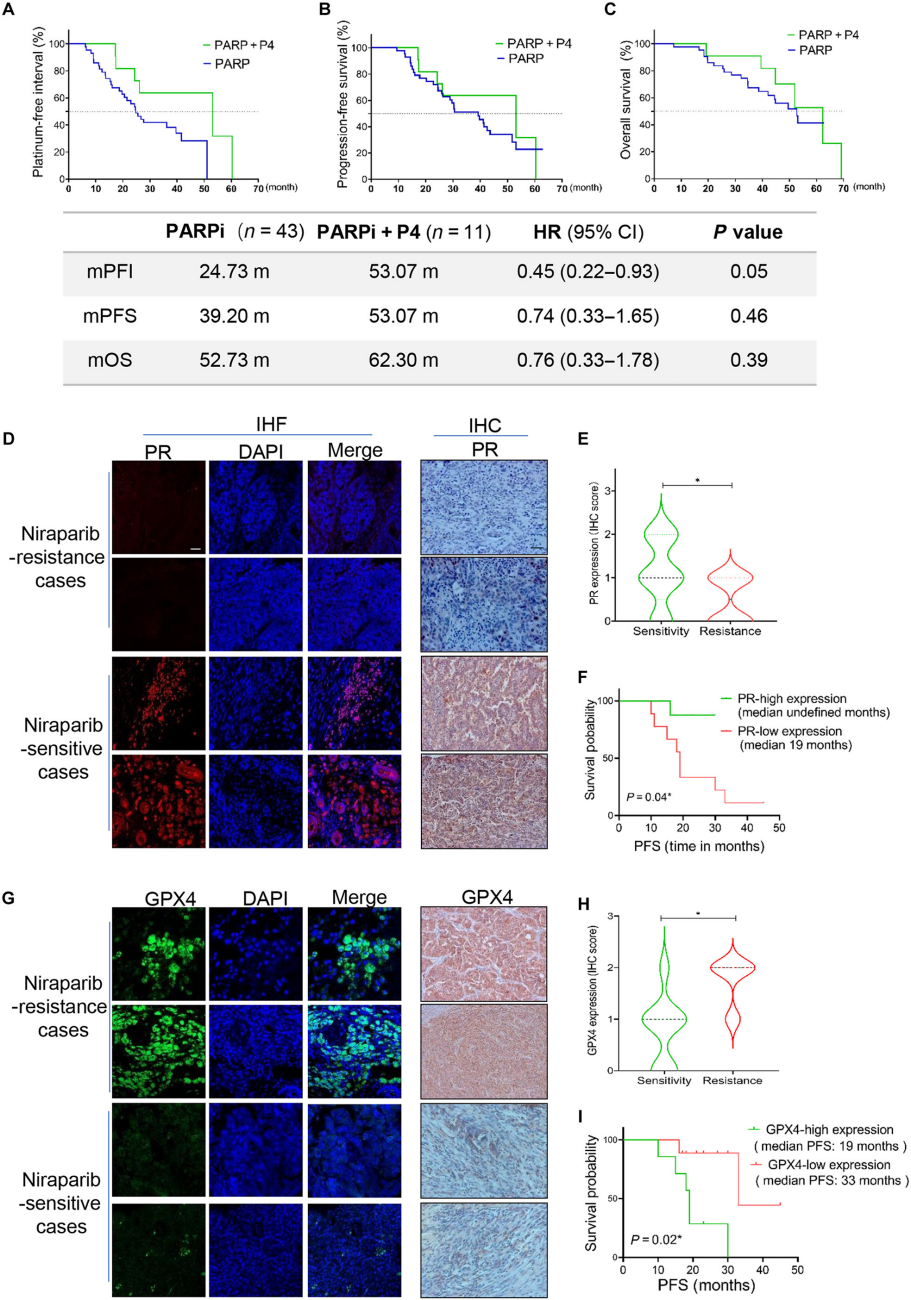
P4 enhances PARPi efficacy in ovarian cancer second-line maintenance therapy, and PR-high/GPX4-low expression was linked to better prognosis and higher PARPi sensitivity. (A) PFI, (B) PFS, and (C) OS of patients with ovarian cancer received second-line maintenance therapy with or without concomitant P4 therapy. IF and IHC test the PR (D and E) and GPX4 (G and H) levels in PARPi-sensitive and -resistant ovarian cancer tissues. (F and I) PFS of patients with ovarian cancer administered with PARPi as maintenance therapy with different levels of PR/GPX4 expression. *P < 0.05. Scale bar, 10 μm. CI, confidence interval; HR, hazard ratio.

To further explore the clinical application, we enrolled a total of 54 patients diagnosed with recurrence ovarian cancer who received PARPi as second-line maintenance therapy. Among them, 11 patients received P4 (medroxyprogesterone acetate) as a concomitant treatment for 1 to 3 months. The patient characteristics are displayed in Table 3. Kaplan–Meier survival analysis found that survival in the PARPi + P4 group was higher than that in the PARPi group: the median progression free interval (mPFI) was 53.07 months versus 24.73 months, the median progression free survival (mPFS) was 53.07 months versus 39.20 months, the median overall survival (mOS) was 62.30 months versus 52.73 months (Fig. 7A to C). IHC and IF showed the higher PR expression (Fig. 7D and E) and lower GPX4 expression (Fig. 7G and H) in PARPi-sensitive cases (*n* = 9) compared with PARPi-resistant cases (*n* = 7) (Table S2). High expression of PR (Fig. 7F) and low expression of GPX4 (Fig. 7I) were associated with longer survival.

**Table 3.** Clinical characteristics of recurrence patients with ovarian cancer (*n* = 54)

Clinical characteristics	PARPi (*n* = 43)	PARPi + P4 (*n* = 11)	*P*
Age (years)	58.56 ± 7.79	56.64 ± 10.5	0.500
BMI (kg/m^2^)	23.38 ± 3.24	22.44 ± 3.23	0.390
Total pregnancies	2.90 ± 1.57	2.90 ± 1.10	0.993
Childbirth	1.86 ± 0.87	1.30 ± 0.48	0.058
Abortion	1.07 ± 1.24	1.60 ± 1.27	0.232
Histological types			
HGSC	43	11	–
FIGO stages			
III	30	7	0.349
IV	5	3	
Unknown	8	1	
Gene mutations			
BRCA^+^	2	1	0.465
BRCA^−^	12	2	
Unknown	29	8	

BMI, body mass index; FIGO, International Federation of Gynecology and Obstetrics.

## Discussion

In this study, we found that P4 enhanced the efficiency of PARPis and prolonged survival in ovarian cancer. P4 acted synergistically with niraparib in ovarian cancer cells regardless of BRCA mutation status, and these synergistic effects were more pronounced in wild-type BRCA ovarian cancer cells. At the optimized mass ratios of 1:1, the addition of P4 to niraparib significantly inhibited cell viability and reduced IC_50_ by 0.5:1- to 4:1-fold compared with niraparib alone in ovarian cancer cell lines with or without *BRCA* mutation. The combination therapy significantly decreased colony formation and metastasis in both *BRCA*^WT^ and *BRCA2*^Mut^ cells compared with either niraparib or P4 alone. Notably, testing on the patient-derived organoids confirmed the synergistic effects with ED_50_ < 1 in both niraparib-sensitive and -resistant cases. The synergistic effect of P4 on niraparib is not limited to type 2 high-grade serous ovarian cancer cells. Two type 1 EOC cells, SKOV3 and A2780, are also sensitized by P4 for niraparib cytotoxicity. SKOV3 has characteristics of clear cell adenocarcinomas [51] with microsatellite instability and carries *ARID1A* and *MLH1* mutations. Molecular characterization of A2780 cells also showed a non-HGSC profile with *ARID1A*, *BRAF*, *PIK3CA*, and *PTEN* mutations, microsatellite instability, and wild-type TP53 [51–53].

P4, a low-toxicity therapeutic that targets various key pathways, has been used for over 80 years [54–56] and is crucial for cancer treatment and prevention [41,57]. In this study, niraparib plus P4 induced serious DNA damage and apoptosis in ovarian cancer cells and tumor tissues. It should be noted that DNA damage stress is an important determinant of PARPis’ sensitivity. Niraparib is a highly selective inhibitor of PARP-1 and PARP-2, which are sensors of DNA damage that are most active during the S phase of the cell cycle [58–60]. These findings suggested that P4 may enhance the effects of PARPis by causing DNA damage and inducing cell death.

Ferroptosis is involved in the development and treatment response of various types of tumors [61–63]. In this study, P4 enhances the activity of niraparib in ovarian cancer by promoting ferroptosis through up-regulated lipid oxidation and POA production. Taken together, P4 enhances niraparib activity synergistically in ovarian cancer by promoting SCD1-mediated fatty acid oxidation and ferroptosis. EOC is characterized by high ROS stress and DNA damage loads with a saturation of DNA damage repair machineries, especially in the HRD high-grade serous carcinoma [64]. This saturated ROS/DNA damage load make ovarian cancer sensitive to the alkylating agents such as cisplatin that cause DNA replication catastrophe. The oversaturated DNA repair makes HRD ovarian cancer vulnerable to PARPis, which cause DNA repair catastrophe [65]. Adding P4 to PARPi deteriorates the ROS and DNA damage stress, overwhelms the DNA repair capacity, and kicks the cell into programed death.

Owing to the defective apoptosis due to p53 loss, overstressed HGSC cells undergo different nonapoptotic ways of death. In the precancerous state, P4 induces necroptosis of p53-mutated cells in the fallopian tube epithelium [41]. In therapeutic prevention model, P4 acts through the adjacent fibroblasts to deliver paracrine inflammatory stress to cancer cells, leading to pyroptosis [42]. The present study unveils the third way of cell death induced by P4, ferroptosis, by enhancing PARPi-induced ROS stress and DNA damage from SCD1-mediated lipid peroxidation.

## Materials and Methods

### Cell culture

The human type 2 EOC cell lines (HGSC, PEO1 cells, BRCA2-mutated/OVCAR3 BRCA wild-type cells) and type 1 EOC cell lines (SKOV3/clear cell carcinoma, A2780/endometroid carcinoma) [66,67] as well as the mouse-derived ovarian cancer ID8 cell line were obtained from the Cancer Institute, Central South University. PEO1 and ID8 cells were cultured in DMEM high-glucose medium (Gibco, Life Technologies, Eugene, OR, USA) containing 10% FBS (Menlo Park, CA, USA), penicillin (100 μg/ml), and streptomycin (100 U/ml). OVCAR3, SKOV3, and A2780 cells were cultured in RPMI 1640 (Gibco, Life Technologies) containing 10% FBS, penicillin (100 μg/ml), and streptomycin (100 U/ml). All cell lines were cultured at 37 ˚C with 5% CO_2_.

### Cell Counting Kit-8 test

Ovarian cancer cells were seeded into 96-well plates at 5 × 10^3^ per well, cultured for 24 h, and then treated with vehicle control, niraparib, P4 (P8783, Sigma-Aldrich) plus niraparib or P4 for 48 h. Then, 10 μl of the CCK8 reagent (KOO9-100, Zeta-Life) was added, and cells were incubated at 37 °C with 5% CO_2_ in the dark for 2 h. Optical density was measured at 450 nm on a spectrophotometer. On the basis of the manufacturer’s instructions, CIs at indicated fraction affected levels were calculated with the CompuSyn software by the Chou–Talalay method with nonconstant ratio combinations. The IC_50_ and CI values were calculated.

### Colony formation assay

PEO1 and OVCAR3 cells were seeded into a 6-cm dish at 1 × 10^3^ per dish and cultured for 24 h. Then, the cells were administered with niraparib (10 μM), P4 (10 μM), niraparib + P4, or vehicle control for 48 h. After formation, cell colonies were fixed with 95% alcohol for 30 min, stained with 1% crystal violet for 1.5 h, and washed with water. The colony formation rate was determined after drying.

### Wound healing assay

PEO1, OVCAR3, SKOV3, and A2780 cells were seeded into 6-well plates at 5 × 10^5 ^per well. When the cells were ~90% confluent, using a sterile 10 μl pipette tip, the middle of the well was scratched. Next, the cells were administered with vehicle control niraparib (10 μM), P4 (10 μM), or niraparib + P4 for 48 h. Images were acquired at 0, 24, and 48 h after drug administration, and the healing area on the scratch was calculated with the ImageJ software.

### Transwell migration assay

PEO1, OVCAR3, SKOV3, and A2780 cells were seeded into a Transwell chamber at 5 × 10^5^ per well. After 6 h of culture, the cells were administered with niraparib (10 μM), P4 (10 μM), niraparib + P4, or vehicle control for 48 h. Then, the membranes with cells were fixed with 4% paraformaldehyde for 40 min and stained with 0.1% crystal violet solution for 20 min. Cells on the inner membrane were wiped with a cotton swab, and the chamber was washed with phosphate-buffered saline (PBS) to remove the excess staining solution. After drying, the chambers were imaged, and the numbers of migrated cells were determined.

### Ovarian cancer organoid model

Cut fresh ovarian cancer tissue into 1- to 2-mm^3^ fragments and dissociate at 37 °C for 1 to 2 h. Pass the dissociated cell suspension through a 70-mesh sieve; centrifuge and discard the supernatant. Mix the cell suspension with matrix adhesive, drop 30 μl of matrix adhesive in the center of the culture dish, place it in the incubator for solidification, and then add 250 μl of ovarian cancer organoid culture medium to ensure that the matrix adhesive is completely covered. Incubate in a 37 °C with 5% CO_2_ incubator.

### Flow cytometry

Cells seeded at 1 × 10^6^ per well into 6-well plates were administered with niraparib (10 μM), P4 (10 μM), niraparib + P4, or vehicle control. Cells were collected by trypsinization and centrifugation. The annexin V/propidium iodide (PI) double-staining kit (Nanjing KeyGen Biotech Co. Ltd.) was used to determine the proportion of apoptotic cells by flow cytometry.

### Mouse ovarian in situ tumorigenesis model and intraperitoneal tumorigenesis model experiment

Female C57BL/6 and BALB-nude mice at 6 to 7 weeks of age were purchased from SLA Laboratory. Female C57BL/6 mice were intraperitoneally injected with 1 × 10^5^/ml of ID8 cells, and female BALB-nude mice were injected 2 × 10^5^/ml of OVCAR3 cells into ovarian–tubal intrabursally or peritoneally. Then, the mice were divided into 4 groups (*n* = 8 per group), including vehicle, P4 (H33020828, Zhejiang Xianju Pharmaceutical; 5 mg/kg by intramuscular injection) [41,42] , niraparib (niraparib at 50 mg/kg by gavage) [68,69], and P4 plus niraparib (P4 at 5 mg/kg and niraparib at 50 mg/kg) groups. Therapy was started 10 d after tumor cells injection. Mice were continuously treated 3 times per week, and animal body weights and ascites were observed. Mice were euthanized after 4 weeks of treatment, and tumor sizes, volumes, and tumor invasion and metastasis were recorded. Mouse tumors were fixed with 10% formalin, embedded in paraffin, and cut into 3-μm sections. Survival analysis of tumor-bearing mice after treatment with control, P4, niraparib, and P4 plus niraparib based on the methods mentioned above were also performed (*n* = 8 per group). The study protocol was approved by the Animal Ethics Committee of Hunan Cancer Hospital.

### Immunofluorescene and immunohistochemistry

The tissue sections or adherent cells were washed with PBS after treatment, fixed with 4% paraformaldehyde, and permeabilized with 0.1% Triton X-100 in PBS. The samples were incubated with primary antibodies targeting γ-H2AX ( ab81299, Abcam, Cambridge, MA, USA; 1:200), SCD1 (ab19862, Abcam; 1:200), Ki67 (ab16667, Abcam; 1:500), GPX4 (ab125066, Abcam; 1:200), and PR (ab2765, Abcam; 1:200), respectively, overnight at 4 °C. IF was followed by incubation with fluorescently labeled secondary antibodies (A11034, Invitrogen, Eugene, OR, USA; 1:1,000) in the dark for 1 h. Then, counterstaining was performed with 4′,6-diamidino-2-phenylindole (DAPI). Cell slides were analyzed by laser scanning confocal microscopy. IHC incubated with enhanced enzyme-labeled goat anti-mouse/rabbit immunoglobulin G (AWS0003a/AWS0002a, Abiowell; 1:1,000) at room temperature for 20 min, with subsequent staining with the DAB (3,3′-diaminobenzidine) kit (CWO125M, CWBIO).

### Western blot

Proteins from ovarian cancer cells were extracted with radioimmunoprecipitation assay lysis buffer, and protein concentrations were determined with the BCA (bicinchoninic acid) kit. Each sample was loaded at 30 μg, subjected to SDS-polyacrylamide gel electrophoresis and transferred onto polyvinylidene difluoride membranes (Millipore, Burlington, MA, USA). Polyvinylidene difluoride membranes were blocked with 5% skimmed milk (Biosharp, Hefei, Anhui, China) for 2 h and incubated overnight at 4 °C with primary antibodies against β-actin (ab8227, Abcam; 1:1,000), glyceraldehyde-3-phosphate dehydrogenase (GAPDH; ab8245, Abcam; 1:1,000), γ-H2AX (ab81299, Abcam; 1:1,000), GPX4 (ab125066, Abcam; 1:1,000), and SCD1 (ab19862, Abcam; 1:1,000). Bound antibodies were then detected with horseradish-peroxidase-conjugated secondary antibodies (AWS0003a, Abiowell; 1:1,000). The ECL Western blotting substrate (Advansta, Munich, Germany) was used for visualization.

### Metabonomics/metabolism

OVCAR3 cells were administered with vehicle, niraparib, niraparib plus P4, or P4 for 24 h, and then samples were thawed on ice bath to diminish sample degradation. Twenty microliters of plasma/serum was added to a 96-well plate. Then, the plate was transferred to the Eppendorf epMotion Workstation (Eppendorf Inc., Humburg, Germany). Ice-cold methanol (180 μl) with partial internal standards was automatically added to each sample and vortexed vigorously for 5 min. The plate was centrifuged at 4,000*g* for 30 min (Allegra X-15R, Beckman Coulter Inc., Indianapolis, IN, USA). Then, the plate was returned back to the workstation. Forty microliters of supernatant was transferred to a clean 96-well plate, and 20 μl of freshly prepared derivative reagents was added to each well. The plate was sealed, and the derivatization was carried out at 30 °C for 60 min. After derivatization, 420 μl of ice-cold acetonitrile was added to dilute the sample. Then, the plate was stored at −20 °C for 20 min and followed by 4,000*g* centrifugation at 4 °C for 30 min.

### ROS test

OVCAR 3, SKOV3, A2780, and PEO1 cells were administered with vehicle, niraparib, niraparib plus P4, or P4 for 24 h; absorbing the culture medium and addition of serum-free medium and ROS (C6827, Invitrogen, USA) probe mixed reagent (10 μM), incubated at 37 °C for 30 min, and washed 3 times with serum-free medium. The collected cells were determined using by flow cytometry.

### BODIPY 581/591 C11 test

OVCAR3, SKOV3, A2780, and PEO1 cells were implanted in laser confocal culture dishes; administered with vehicle, niraparib, niraparib plus P4, or P4 for 24 h, absorbing the culture medium; added BODIPY 581/591 C11 reagent (D3861, Invitrogen, USA; 10 μM); mixed culture medium; incubated at 37 °C for 30 min; absorbed working fluid; added serum-free culture medium after PBS washing; and then analyzed by confocal laser scanning microscopy.

### Quantitative real-time polymerase chain reaction

Total RNA was extracted from OVCAR3, SKOV3, A2780, and PEO1 cells with TRIzol reagent (Ambion Inc., Austin, TX, USA). Total RNA was reverse-transcribed into complementary DNA with PrimeScript RT reagent kit with gDNA Eraser (TaKaRa Biotechnology, Dalian, China), following the manufacturer’s instructions. TB Green Premix Ex Taq II (TaKaRa Biotechnology) was used for PCR on a LightCycle 480 II instrument. All primers were synthesized by Sangon Biotechnology Company (Shanghai, China) and are listed in Table S1. The relative mRNA expression was calculated by the 2^−ΔΔCt^.

### Patient cohort

The retrospective cohort included 54 patients diagnosed with recurrent EOC who received PARPi as maintenance therapy between 2019 and 2021 at Hunan Cancer Hospital; among them, 11 people were taking P4 (megestrol acetate dispersible tablets, H20040001) a concomitant treatment for 1 to 3 months. The inclusion criteria are as follows: (a) platinum sensitivity; (b) the efficacy of primary treatment achieves partial or complete remission; and (c) PARPi was received as maintenance therapy more than 6 months. The exclusion criteria are as follows: (a) without platinum-based chemotherapy; (b) treated with other targeted drugs except PARPi; (c) clinical information missing; (d) missed follow-up. The study protocol was approved by the Institutional Review Board of Hunan Cancer Hospital.

### Statistical analysis

Progression-free survival (PFS) was the time starting from patients’ enrollment until tumor progression or death. Platinum-free interval (PFI) refers to the time from completion of the last platinum-containing chemotherapy course to recurrence. Overall survival (OS) refers to the time from patients’ enrollment to death.All data were obtained from 3 independent replicates, and the results were expressed as means ± SD. Statistical analysis was performed with SPSS version 18.0 or GraphPad. Two-sample *t* test or nonparametric test was used to compare group pairs. The chi-square test was used for categorical data. Kaplan–Meier analysis was performed to assess the prognosis of patients with ovarian cancer, and the log-rank test was performed to compare survival. *P* < 0.05 was considered statistically significant.

## Data Availability

Data will be made available on request.
