# Supplementary material for: Progesterone Enhances Niraparib Efficacy in Ovarian Cancer by Promoting Palmitoleic-Acid-Mediated Ferroptosis
Source: Research (Wash D C). 2024 May 24;7:0371. doi: 10.34133/research.0371 (PMC11116976; doi:10.34133/research.0371)

Figure 2B OVCAR3

β-actin

NC Nira Nira+P4 P4

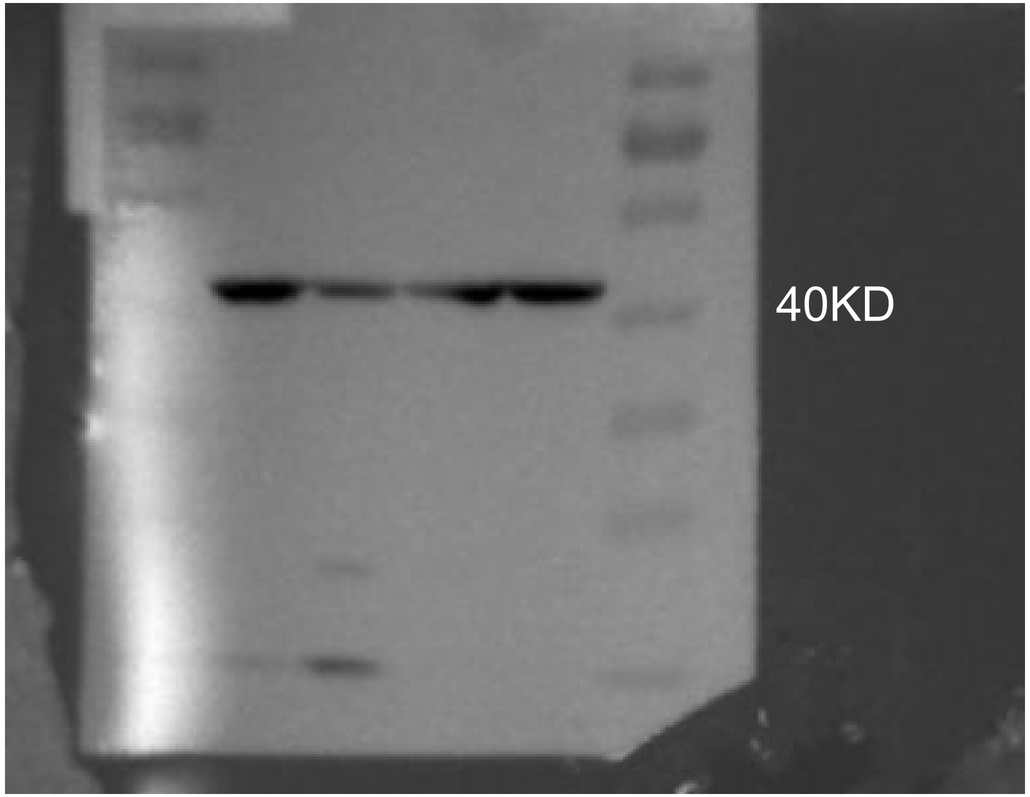

p-γH2AX

NC Nira Nira+P4 P4

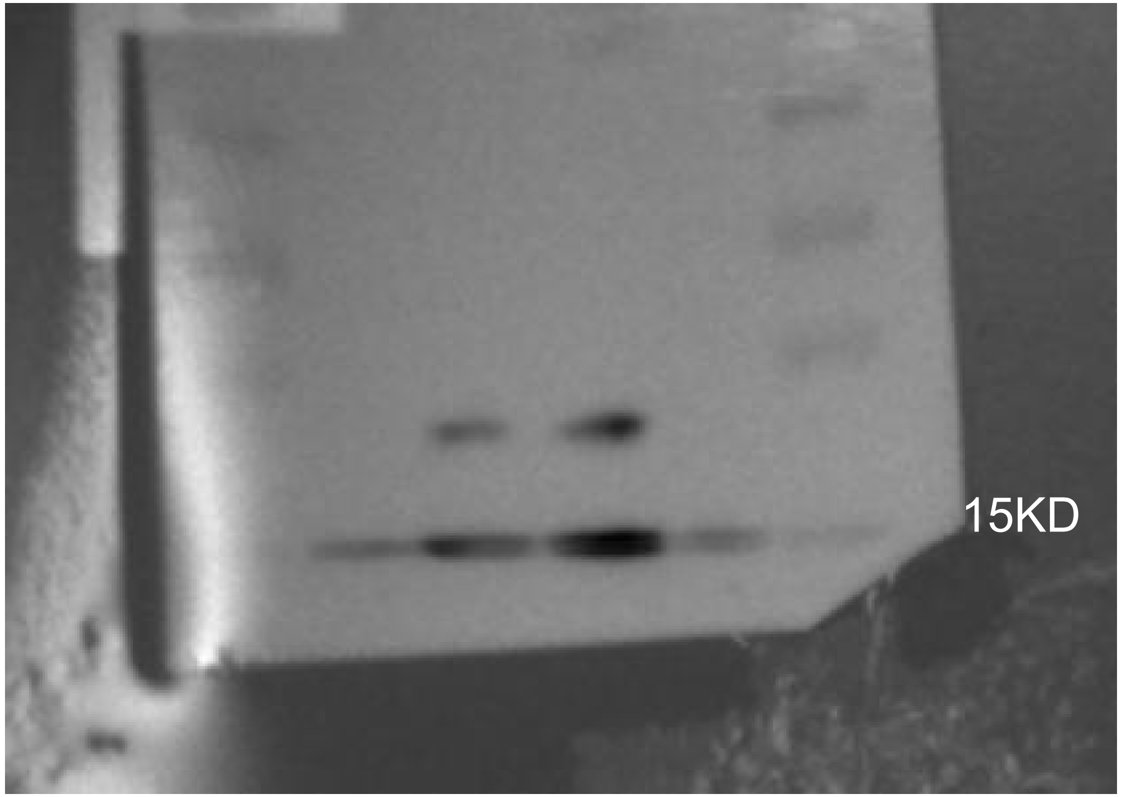

Figure 2B A2780

β-actin

P4 Nira+P4 Nira NC

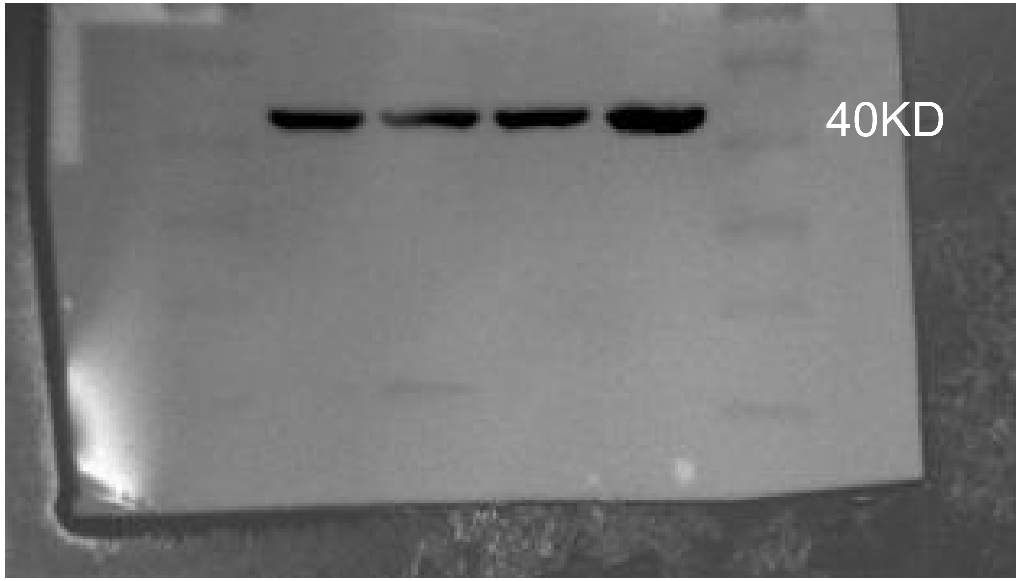

p-γH2AX

P4 Nira+P4 Nira NC

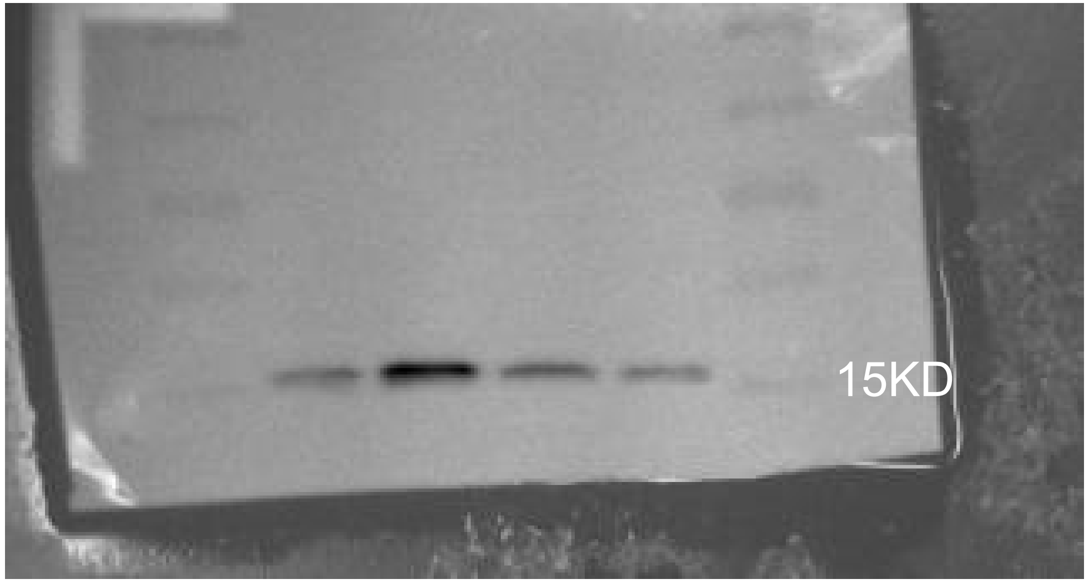

Figure 2B PEO1

β-actin

P4 Nira+P4 Nira NC

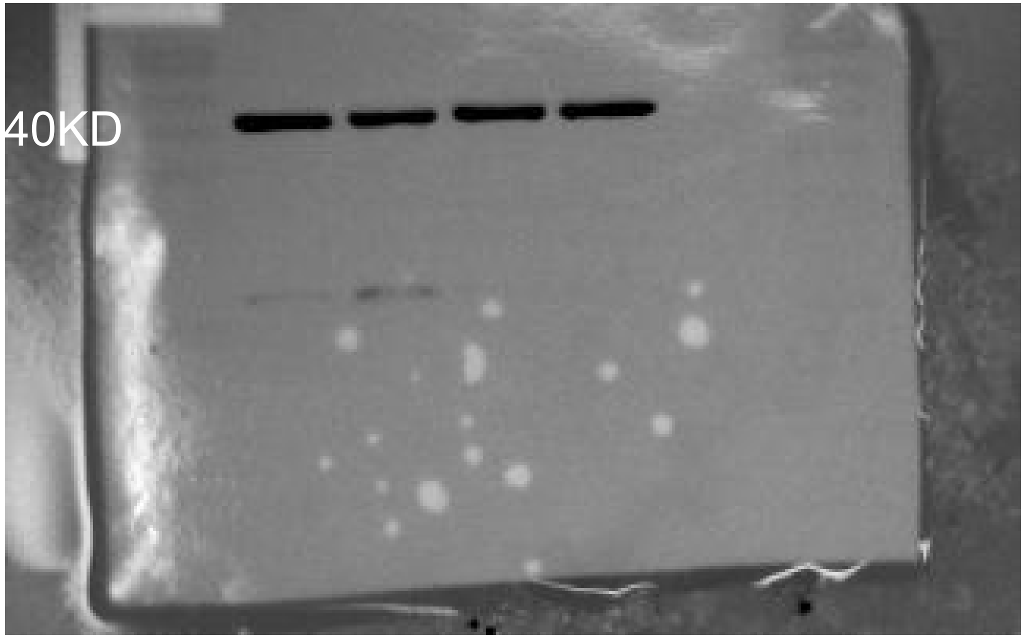

p-γH2AX

P4 Nira+P4 Nira NC

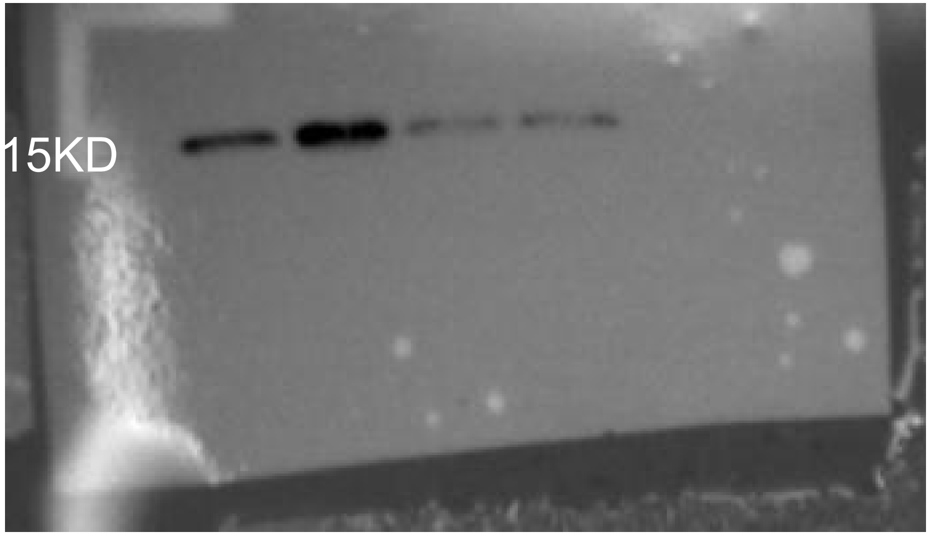

Figure 4F OVCAR3

GAPDH

NC Nira POA+Nira POA

γH2AX

NC Nira POA+Nira POA

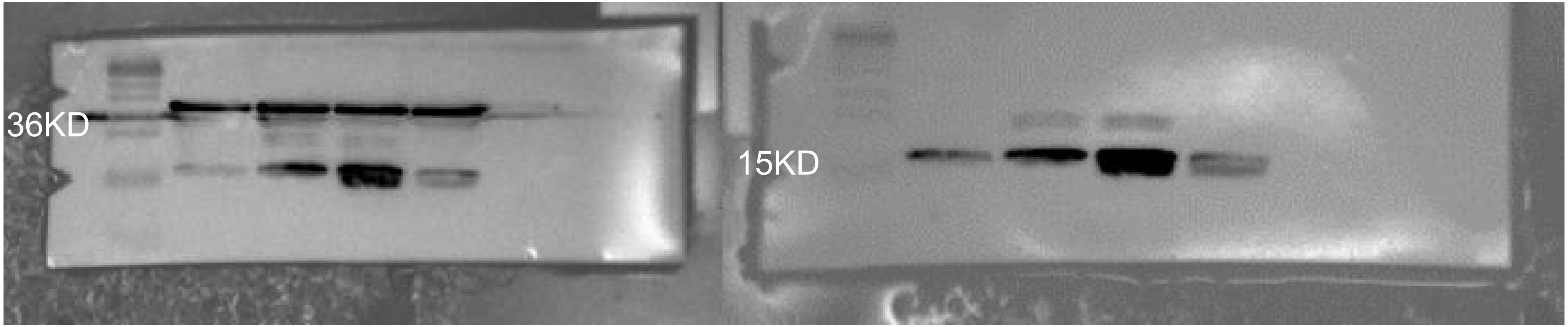

Figure 4F SKOV3

GAPDH

POA POA+Nira Nira NC

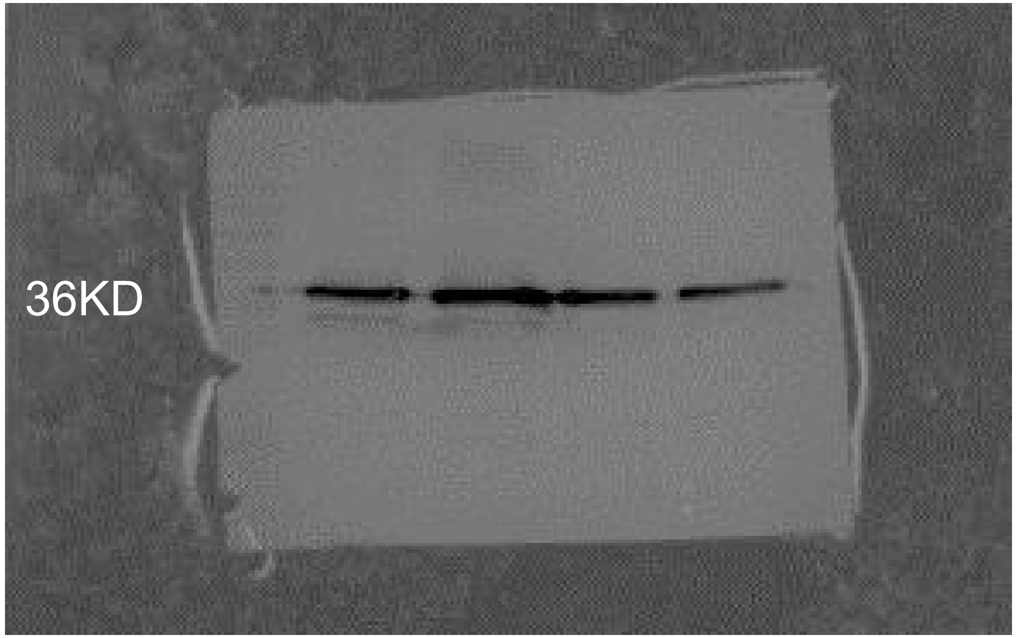

γH2AX

POA POA+Nira Nira NC

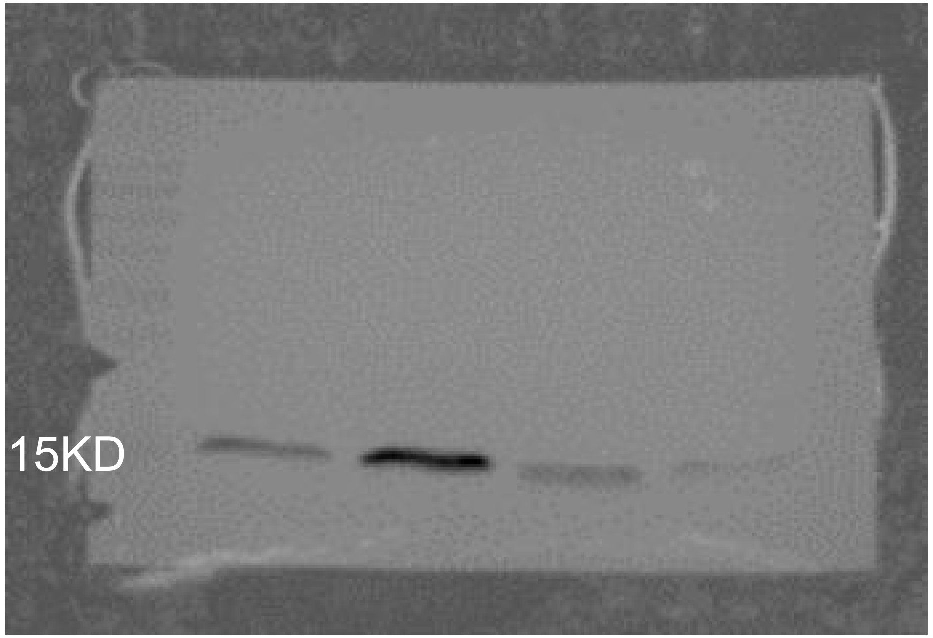

Figure 4H OVCAR3

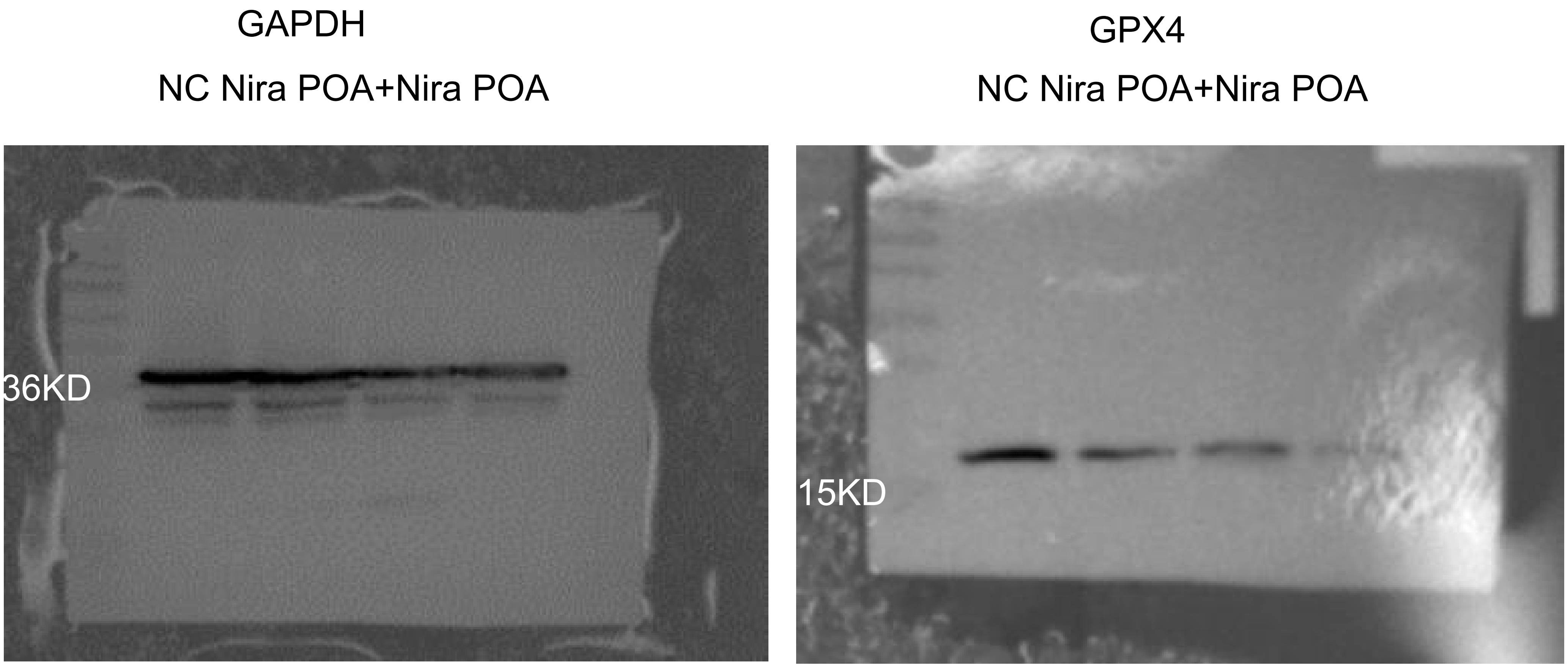

Figure 4H SKOV3

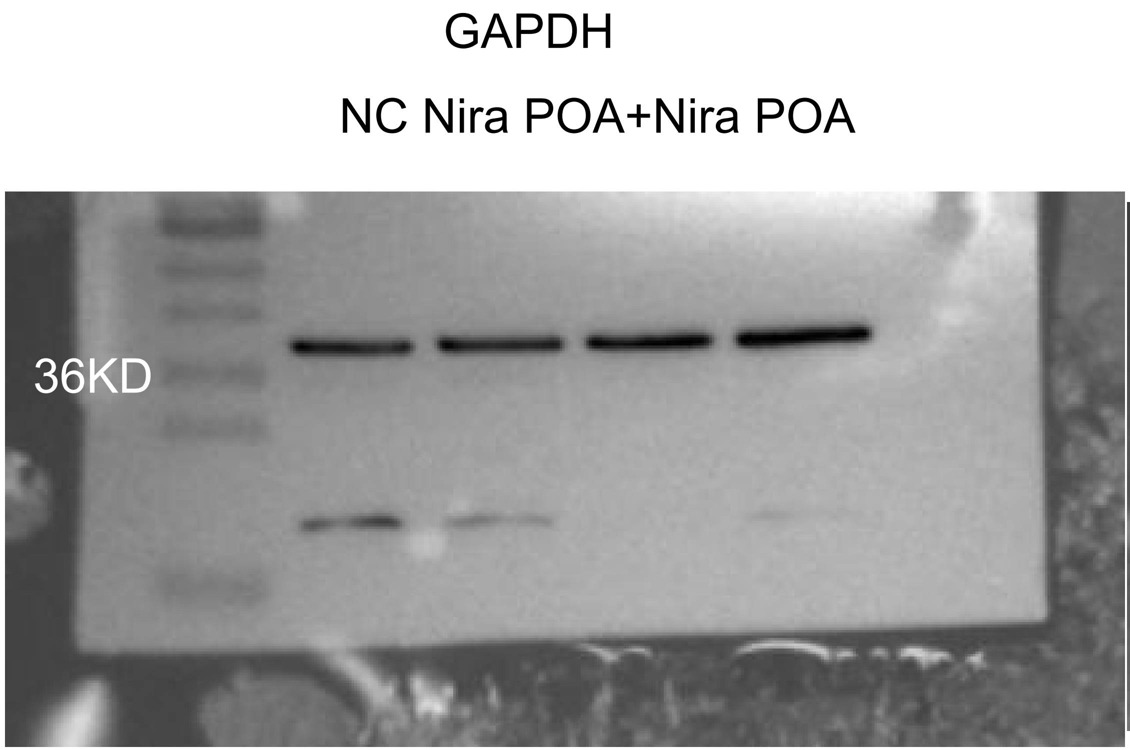

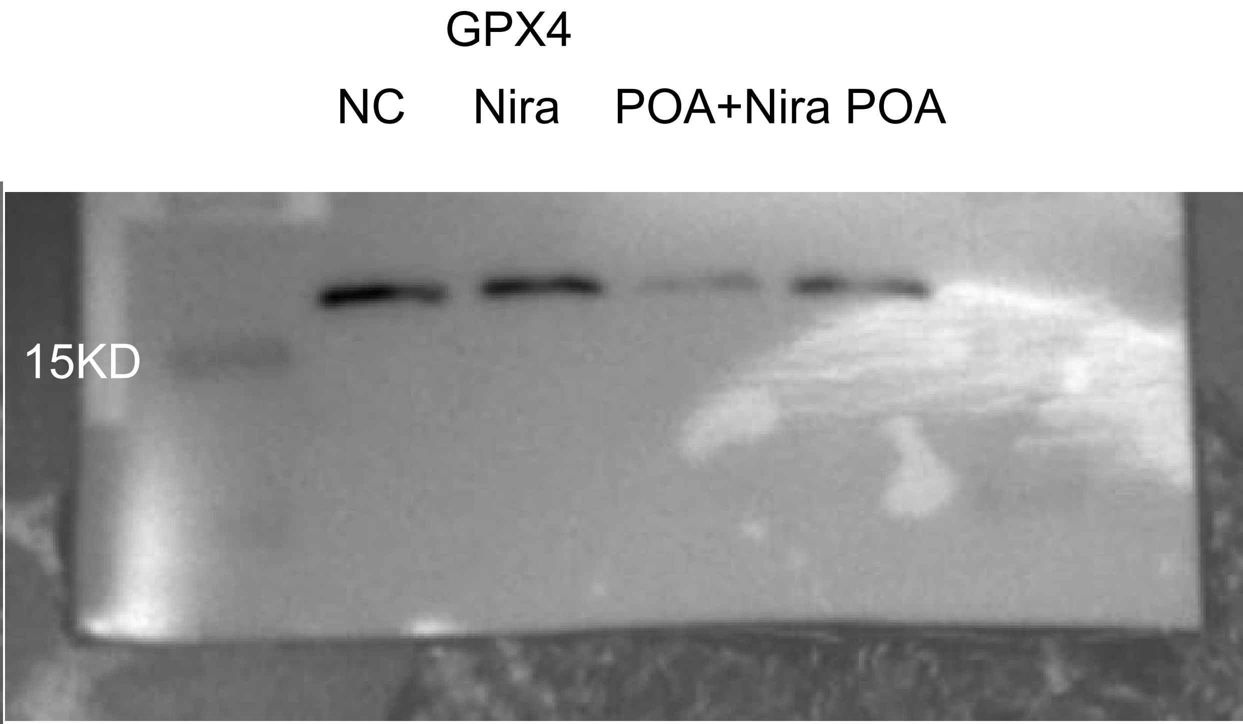

Figure 5C OVCAR3

β-actin

P4 Nira+P4 Nira NC

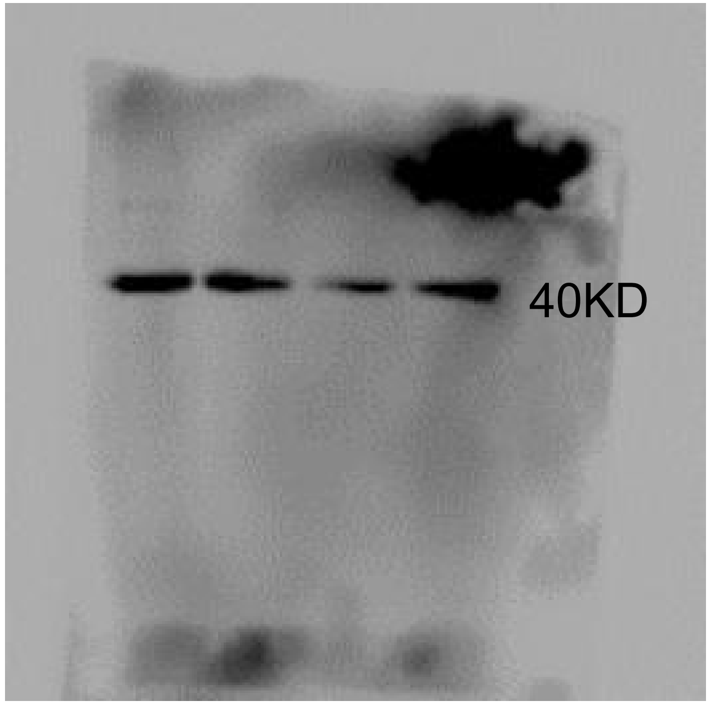

GPX4

P4 Nira+P4 Nira NC

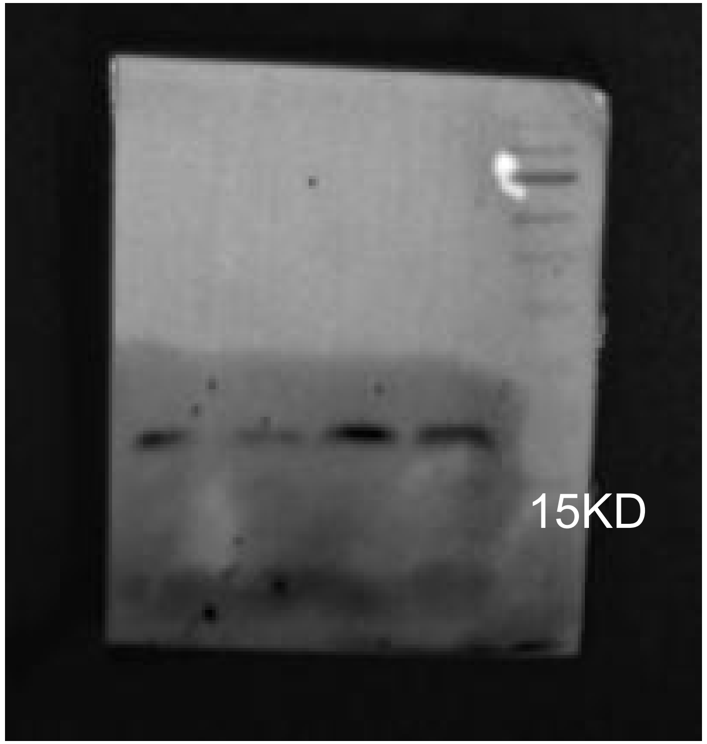

Figure 5C SKOV3

β-actin

NC Nira Nira+P4 P4

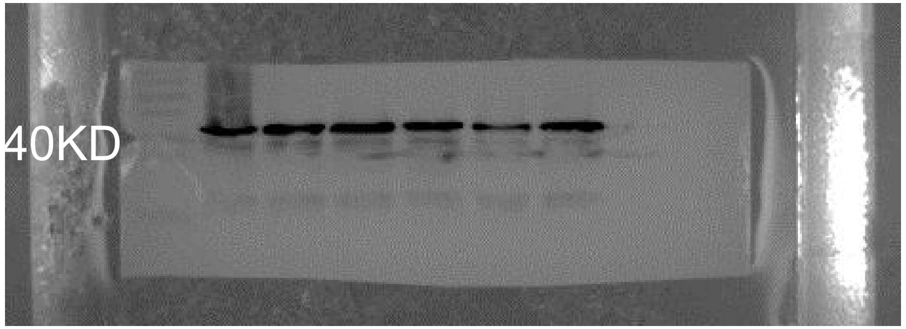

GPX4

NC Nira Nira+P4 P4

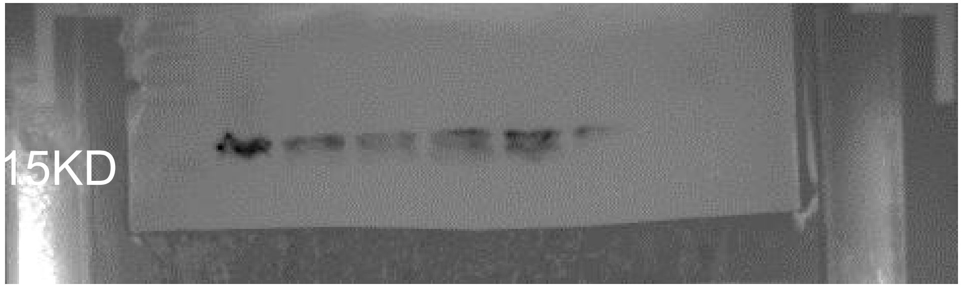

Figure 5D OVCAR3

β-actin

NC Nira+P4 Nira+P4+Lip-1

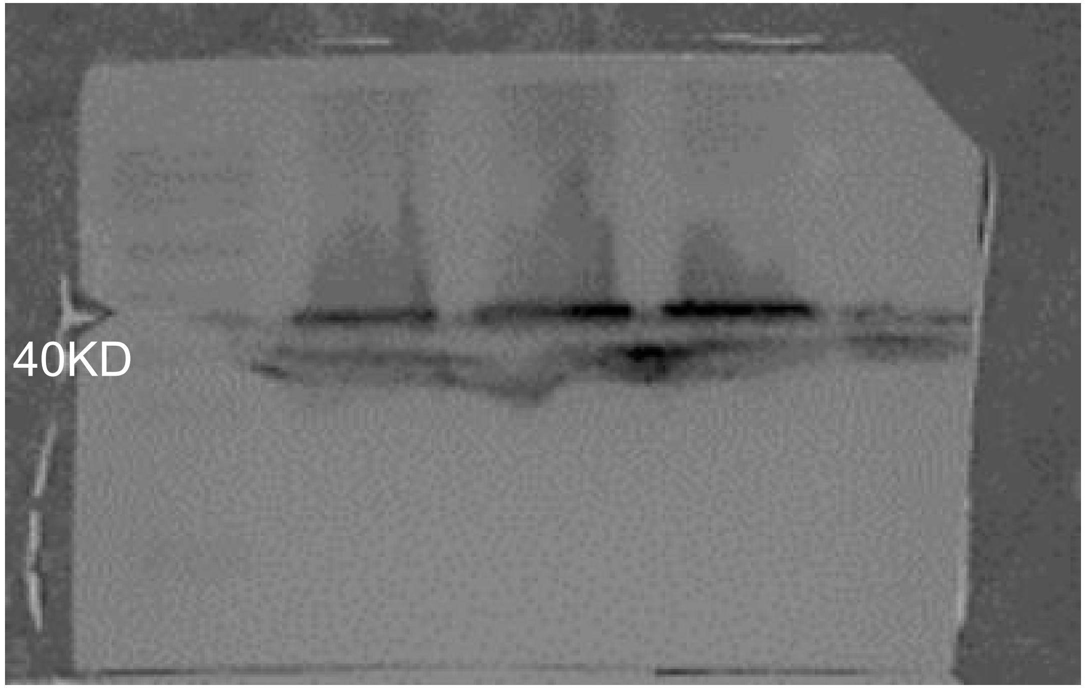

GPX4

NC Nira+P4 Nira+P4+Lip-1

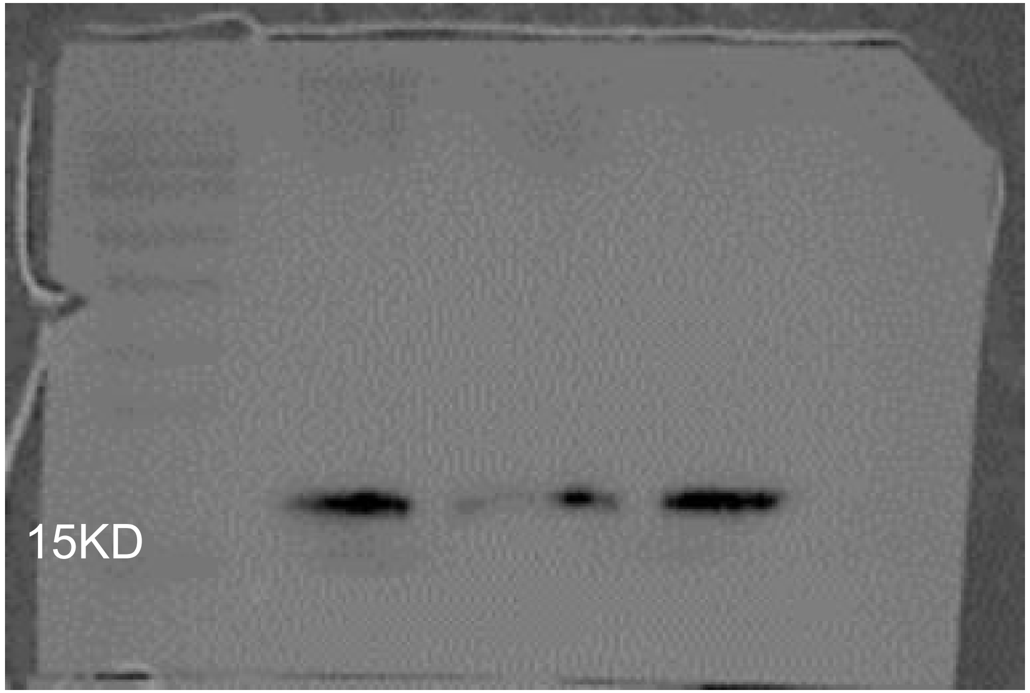

Figure 5D SKOV3

β-actin

NC Nira+P4 Nira+P4+Lip-1 Nira+P4

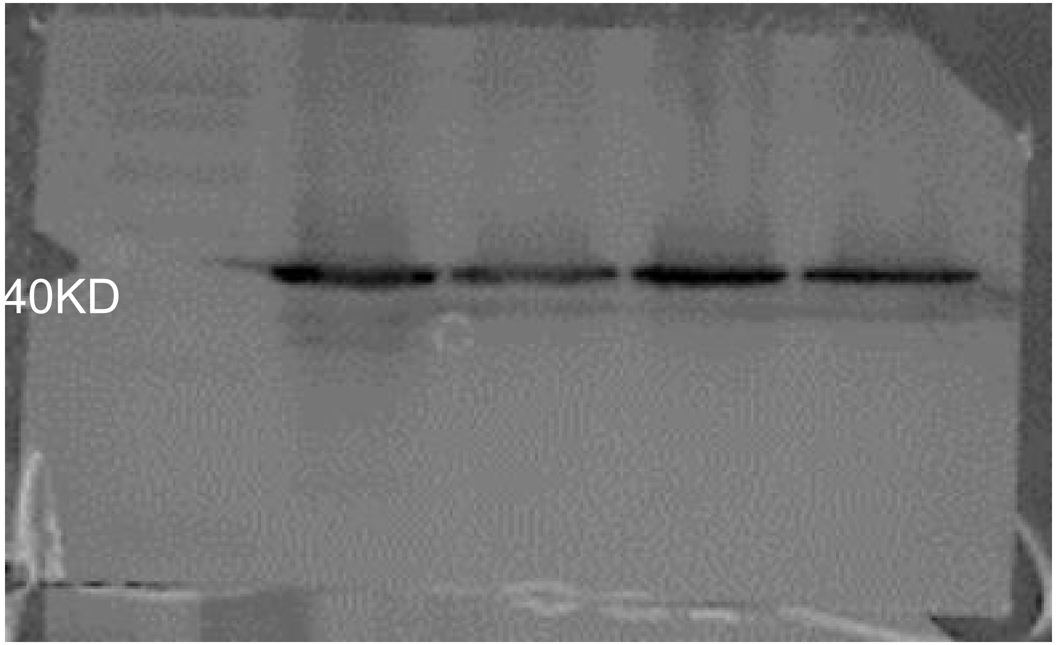

GPX4

NC Nira+P4 Nira+P4+Lip-1 Nira+P4

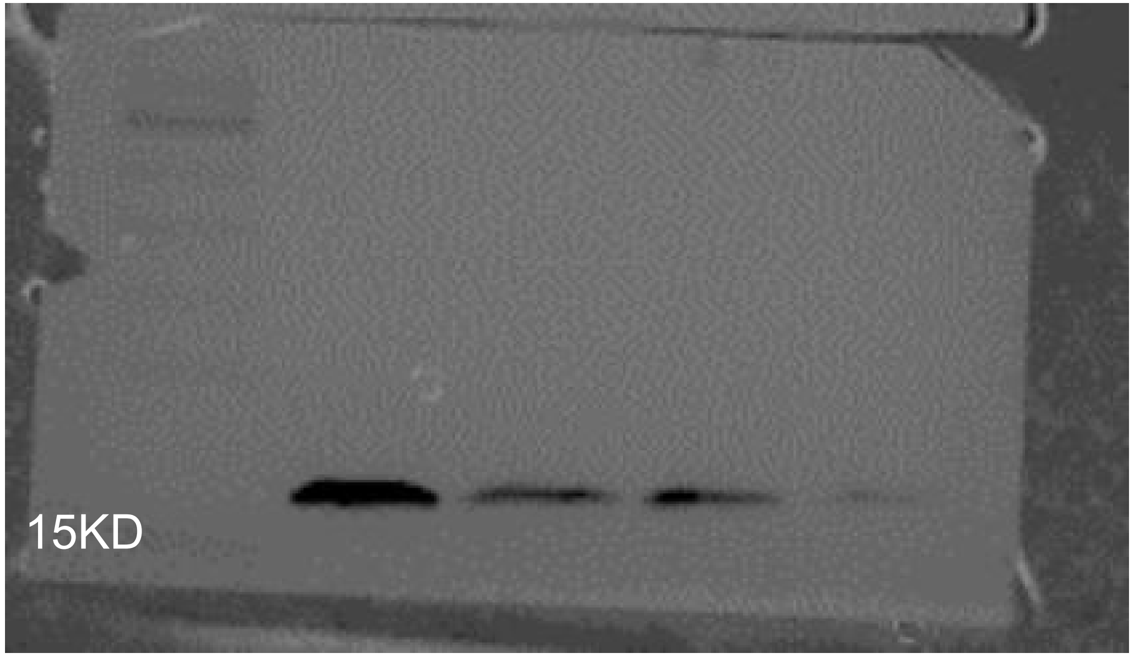

Figure 5E   OVCAR3

GAPDH

NC Nira+P4 Nira+P4+RU486

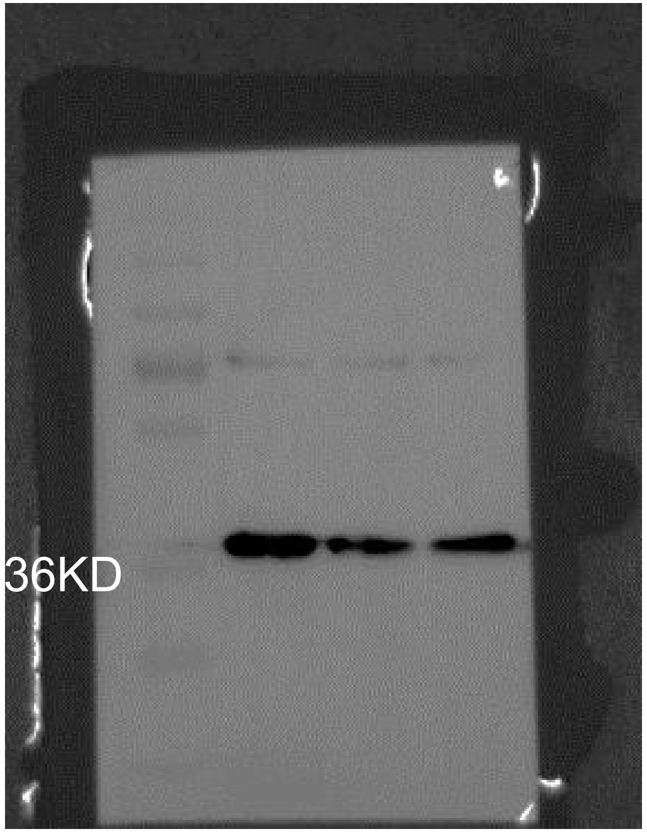

GPX4

NC Nira+P4   Nira+P4+RU486

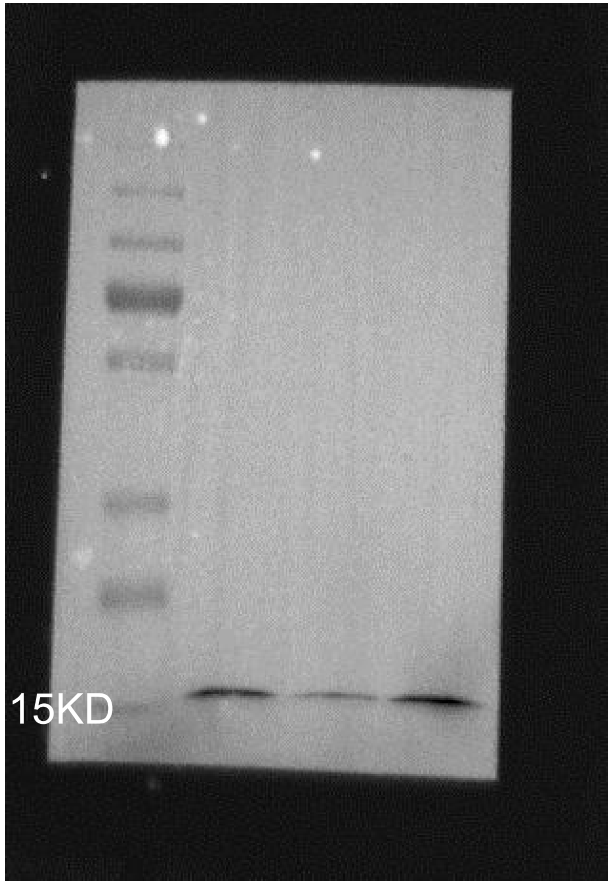

Figure 5E SKOV3

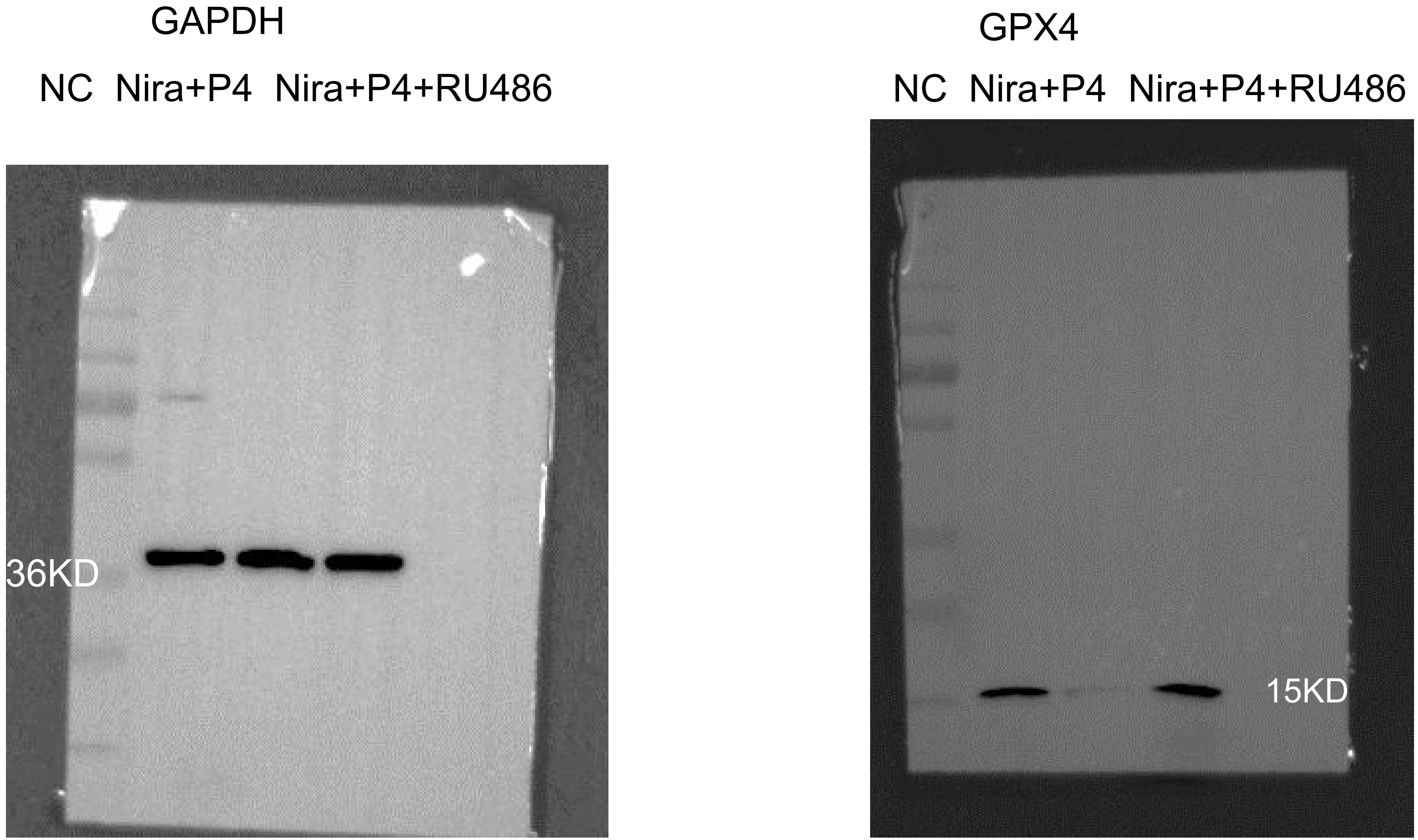

Supplementary Figure 2D OVCAR3

GAPDH

NC Nira Nira+P4 P4

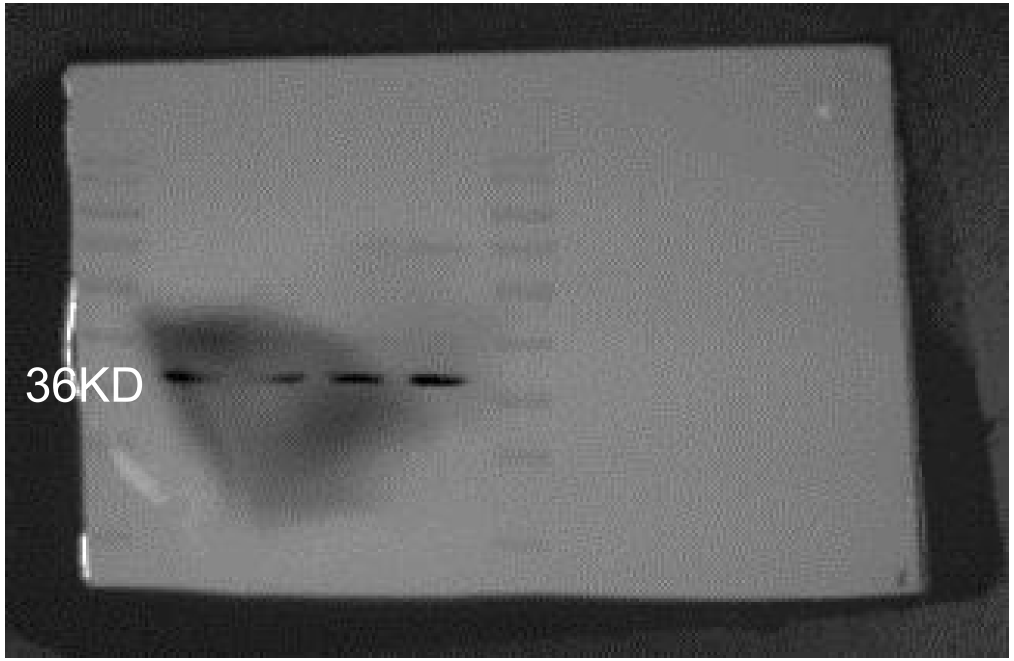

SCD1

NC Nira Nira+P4 P4

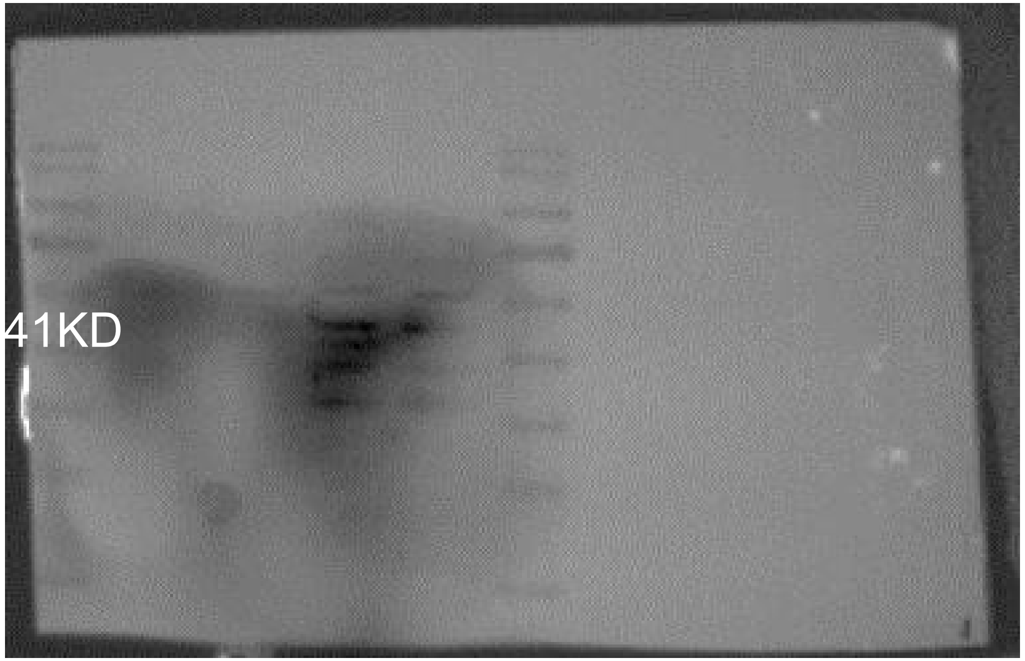

Supplement: Supplementary 1 — Figs. S1 to S4 Tables S1 and S2 [file research.0371.f1.zip › Supplemental WB raw data.pdf]
