# Supplementary material for: Progesterone Enhances Niraparib Efficacy in Ovarian Cancer by Promoting Palmitoleic-Acid-Mediated Ferroptosis
Source: Research (Wash D C). 2024 May 24;7:0371. doi: 10.34133/research.0371 (PMC11116976; doi:10.34133/research.0371)
Supplement: Supplementary 1 — Figs. S1 to S4 Tables S1 and S2 [file research.0371.f1.zip › Supplementary Table1.docx]

| Gene Name |  | Primer sequence (5' -3') |
| --- | --- | --- |
| GAPDH | Forward | GAAGGTGAAGGTCGGAGTC |
|  | Reverse | GAAGATGGTGATGGGATTTC |
| SCD1 | Forward | ACGCTTGTGCCCTGGTATTTCTG |
|  | Reverse | GCATTAAGCACCACAGCATATCGC |
| PCSK9 | Forward | TGGAACCTGGAGCGGATTACCC |
|  | Reverse | GTGGTCACTCTGTATGCTGGTGTC |
| FDFT1 | Forward | GGGCAGTGAAGATTAGGAAAGGG |
|  | Reverse | ATGGGTCTGAGTCGGGGATTCTATG |
| FASN | Forward | GTGGTGGGCTTGGTGAACTGTC |
|  | Reverse | AGGTGCTGCTGAGGTTGGAGAG |
| ASCC2 | Forward | AGGCAGTCAGTCAAGCATCATCAC |
|  | Reverse | CTGGCAGCAGGTCCTTCACTTG |
| DHCR7 | Forward | ACCCAACATTCCCAAAGCCAAGAG |
|  | Reverse | GGCGAACAGCAGTAGGAAGATGAC |
| MVD | Forward | GCCCATCTCTTACCTCAATGCCATC |
|  | Reverse | ACTCAGCCACAGTGTCGTCCAG |
| PER1 | Forward | CCGATGACACCGATGCCAACAG |
|  | Reverse | TCTTGCTGCTCTCAGTGGTCTCC |
| INSIG1 | Forward | GCTGATACCCTTCTGTGAGGAGTTG |
|  | Reverse | TCACTATGGGGCTTTTCAGGAACAC |
| ETV4 | Forward | GTGCCTTTACTCCAGTGCCTATGAC |
|  | Reverse | GGTGCCAGAGGATCTCAGGAAATTC |

**Supplementary Table1.** Primer sequence

GAPDH: Glyceraldehyde-3-phosphate Dehydrogenase, SCD1: Stearoyl-CoA Desaturase-1, PCSK9: Proprotein Convertase Subtilisin/Kexin Type 9,FDFT1: Farnesyl-diphosphate Farnesyltransferase 1, FASN: Fatty Acid Synthase, ASCC2: Activating Signal Cointegrator 1 Complex Subunit 2,DHCR7: 7-Dehydrocholesterol Reductase, MVD: Mevalonate Diphosphate Decarboxylase, PER1: Period Circadian Regulator 1 INSIG1: Insulin Induced Gene 1, ETV4: ETS Variant Transcription Factor 4.
