# Supplementary material for: Progesterone Enhances Niraparib Efficacy in Ovarian Cancer by Promoting Palmitoleic-Acid-Mediated Ferroptosis
Source: Research (Wash D C). 2024 May 24;7:0371. doi: 10.34133/research.0371 (PMC11116976; doi:10.34133/research.0371)
Supplement: Supplementary 1 — Figs. S1 to S4 Tables S1 and S2 [file research.0371.f1.zip › Supplementary Table2.docx]

**Supplementary Table2.** Correlation between PR and GPX4 expression and sensitivity to PARP inhibitors in HGSC patients

| IHC Score | PARPi Sensitive | | PARPi resistance |
| --- | --- | --- | --- |
| PR expression | 7 | 1 | |
| Non-PR expression | 2 | 7 | |
| P Value | 0.015* | | |
|  |  | | |
| GPX4-high expression | 1 | 6 | |
| GPX4-low expression | 8 | 2 | |
| P Value | 0.015* | | |
|  |  | | |

PR expression: IHC scores were1-2; Non-PR expression: IHC score was 0.

GPX4-High expression: IHC scores were 2-4; GPX4-Low expression: IHC score was 0 and 1.
