# Supplementary figures and images for: Progesterone Enhances Niraparib Efficacy in Ovarian Cancer by Promoting Palmitoleic-Acid-Mediated Ferroptosis
Source: Research (Wash D C). 2024 May 24;7:0371. doi: 10.34133/research.0371 (PMC11116976; doi:10.34133/research.0371)

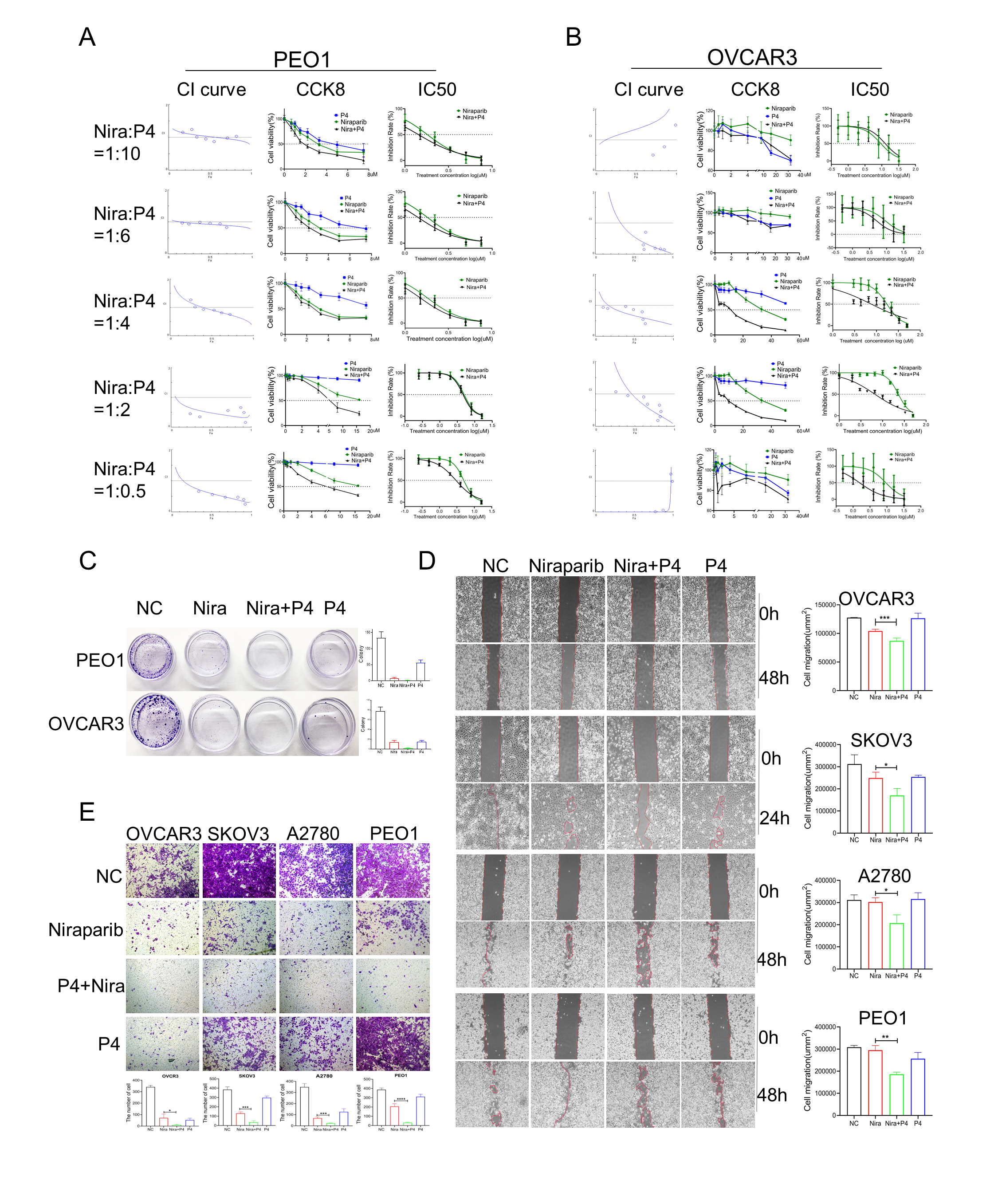

Supplement: Supplementary 1 — Figs. S1 to S4 Tables S1 and S2 [file research.0371.f1.zip › Supplementary Figure 1.TIF]

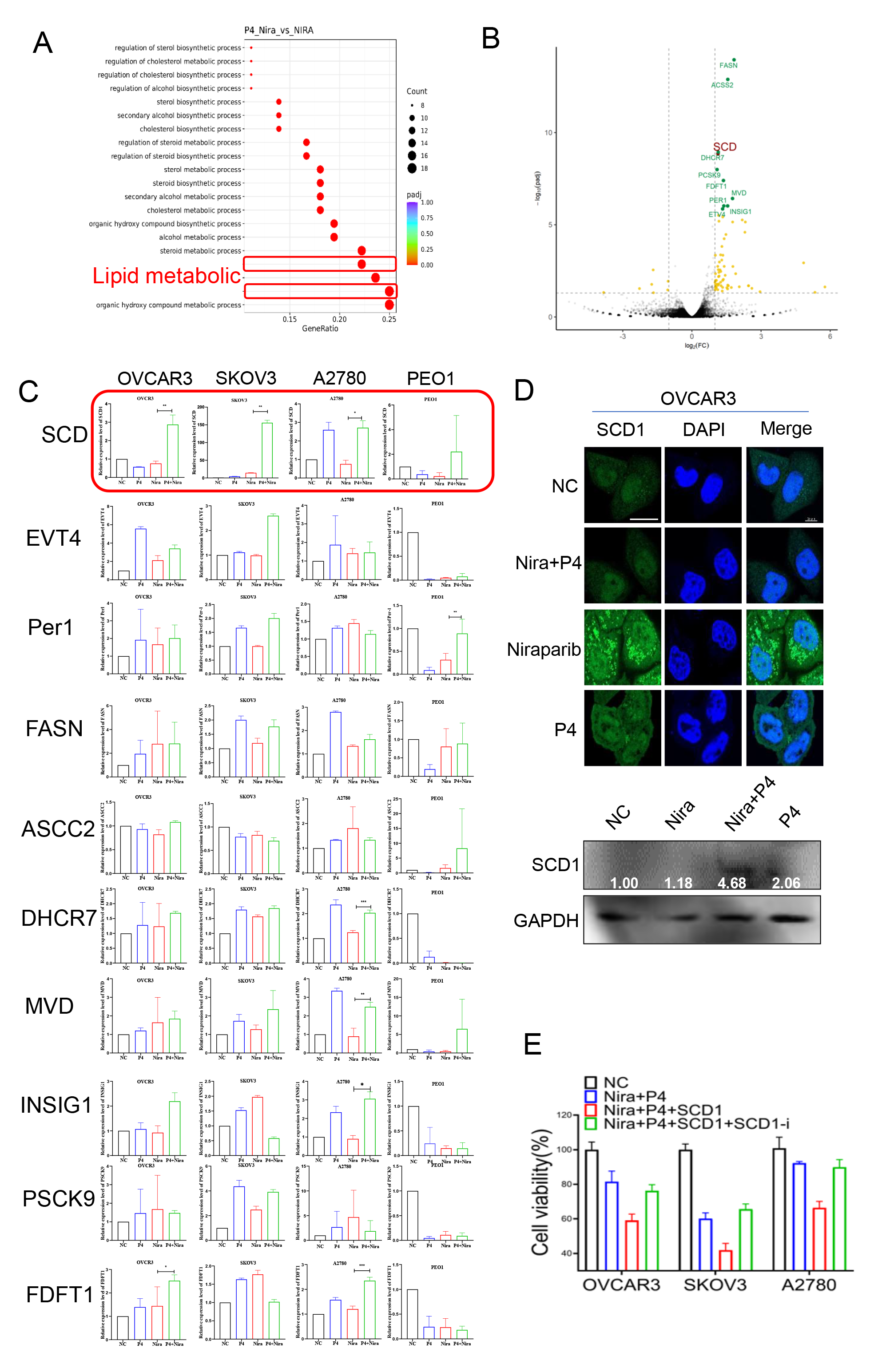

Supplement: Supplementary 1 — Figs. S1 to S4 Tables S1 and S2 [file research.0371.f1.zip › Supplementary Figure 2.TIF]

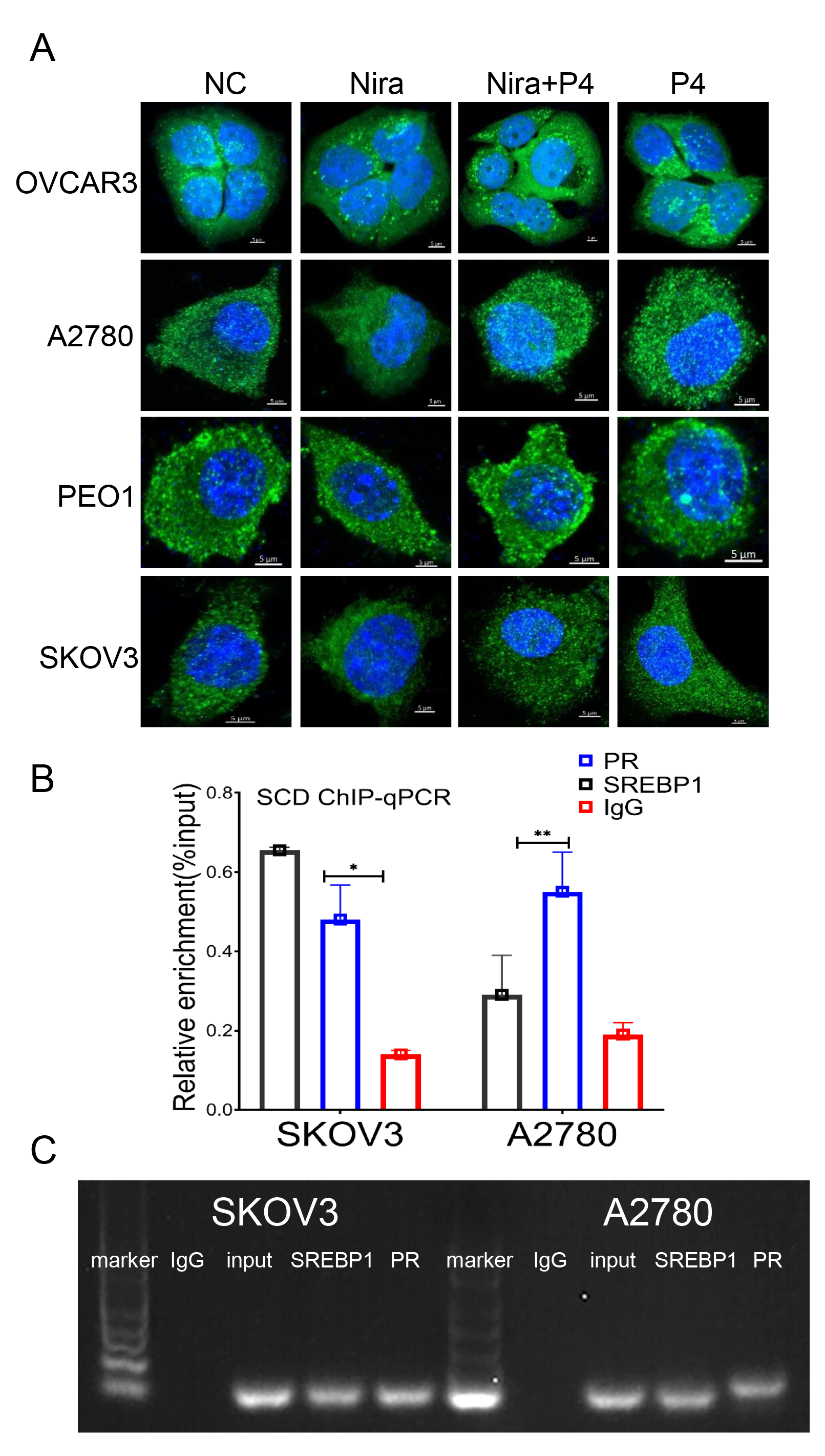

Supplement: Supplementary 1 — Figs. S1 to S4 Tables S1 and S2 [file research.0371.f1.zip › Supplementary Figure 3.TIF]

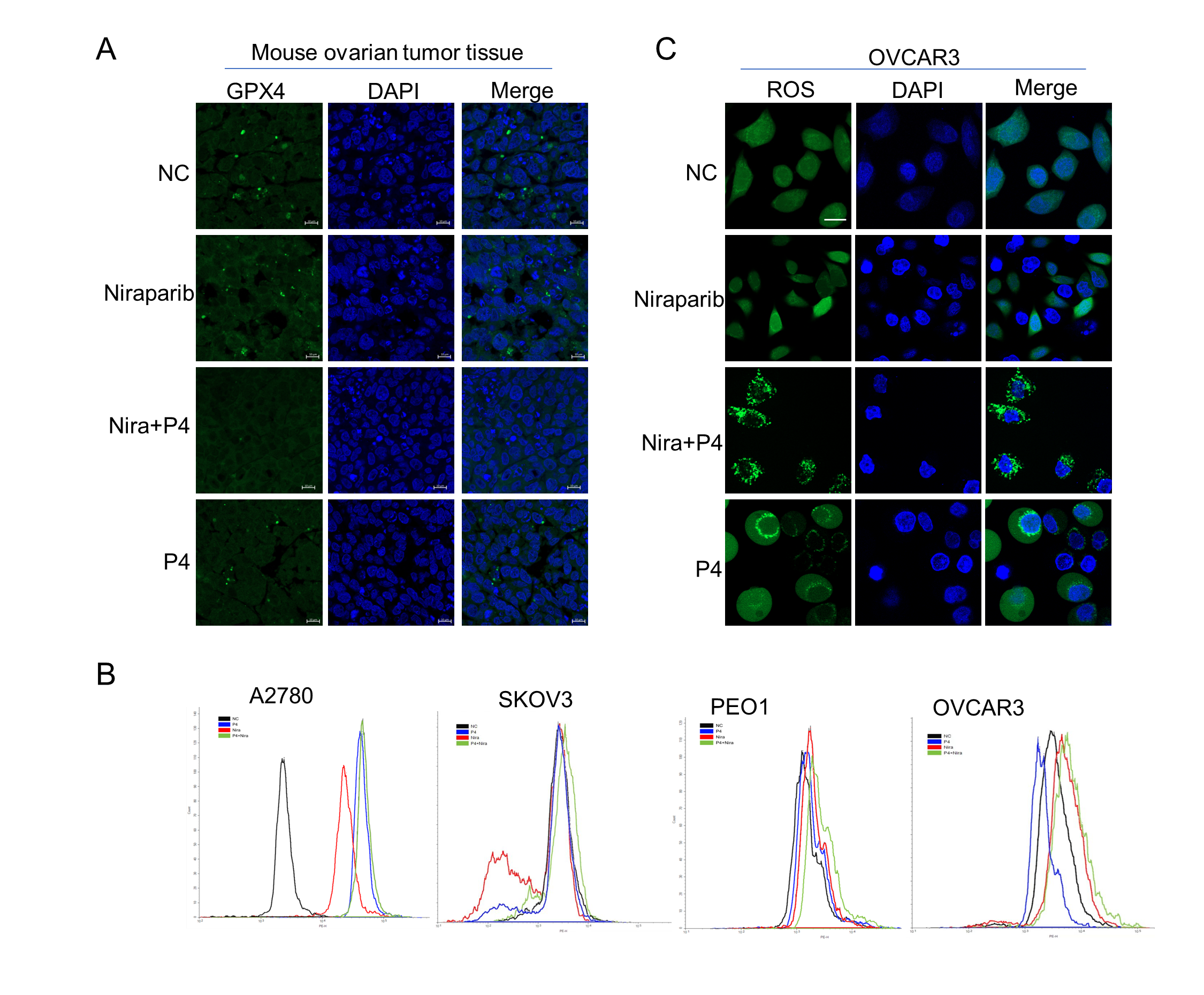

Supplement: Supplementary 1 — Figs. S1 to S4 Tables S1 and S2 [file research.0371.f1.zip › Supplementary Figure 4.TIF]
